# Immune Antibodies and Helminth Products Drive CXCR2-Dependent Macrophage-Myofibroblast Crosstalk to Promote Intestinal Repair

**DOI:** 10.1371/journal.ppat.1004778

**Published:** 2015-03-25

**Authors:** Julia Esser-von Bieren, Beatrice Volpe, Duncan B. Sutherland, Jérôme Bürgi, J. Sjef Verbeek, Benjamin J. Marsland, Joseph F. Urban, Nicola L. Harris

**Affiliations:** 1 Laboratory of Intestinal Immunology, Global Health Institute, School of Life Sciences, École Polytechnique Fédérale de Lausanne (EPFL), Lausanne, Switzerland; 2 Center of Allergy and Environment (ZAUM), member of the German Center for Lung Research (DZL), Technische Universität and Helmholtz Center Munich, Munich, Germany; 3 Laboratory of Cell and Membrane Biology, Global Health Institute, School of Life Sciences, École Polytechnique Fédérale de Lausanne (EPFL), Lausanne, Switzerland; 4 Department of Human Genetics, Leiden University Medical Center, Leiden, The Netherlands; 5 Faculty of Biology and Medicine, University of Lausanne, Service de Pneumologie, Centre Hospitalier Universitaire Vaudois (CHUV), Lausanne, Switzerland; 6 Diet, Genomics, & Immunology Laboratory, Beltsville Human Nutrition Research Center, Agricultural Research Service, United States Department of Agriculture, Beltsville, Maryland, United States of America; University of Manchester, UNITED KINGDOM

## Abstract

Helminth parasites can cause considerable damage when migrating through host tissues, thus making rapid tissue repair imperative to prevent bleeding and bacterial dissemination particularly during enteric infection. However, how protective type 2 responses targeted against these tissue-disruptive multicellular parasites might contribute to homeostatic wound healing in the intestine has remained unclear. Here, we observed that mice lacking antibodies (Aid^-/-^) or activating Fc receptors (Fcrg^-/-^) displayed impaired intestinal repair following infection with the murine helminth *Heligmosomoides polygyrus bakeri (Hpb)*, whilst transfer of immune serum could partially restore chemokine production and rescue wound healing in Aid^-/-^ mice. Impaired healing was associated with a reduced expression of CXCR2 ligands (CXCL2/3) by macrophages (MΦ) and myofibroblasts (MF) within intestinal lesions. Whilst antibodies and helminths together triggered CXCL2 production by MΦ in vitro via surface FcR engagement, chemokine secretion by intestinal MF was elicited by helminths directly via Fcrg-chain/dectin2 signaling. Blockade of CXCR2 during *Hpb* challenge infection reproduced the delayed wound repair observed in helminth infected Aid^-/-^ and Fcrg^-/-^ mice. Finally, conditioned media from human MΦ stimulated with infective larvae of the helminth *Ascaris suum* together with immune serum, promoted CXCR2-dependent scratch wound closure by human MF in vitro. Collectively our findings suggest that helminths and antibodies instruct a chemokine driven MΦ-MF crosstalk to promote intestinal repair, a capacity that may be harnessed in clinical settings of impaired wound healing.

## Author Summary

To complete their lifecycles, helminth parasites have to migrate through tissues such as the skin, lung, liver and intestine. This migration causes severe tissue damage, resulting in the need for rapid repair to restore the integrity and function of damaged tissues. Protective type 2 immune responses against helminths can repair acute lung damage, but they can also promote liver fibrosis. However, how protective immune mechanisms might contribute to wound healing during enteric nematode infection has remained unclear. Here we show that during a protective antibody response, where helminth larvae are trapped in the intestinal mucosa, macrophages and myofibroblasts secrete chemokines, which promote the repair of helminth-caused lesions. Chemokine secretion by macrophages was triggered by antibodies and helminth products, whilst myofibroblasts produced chemokines directly in response to innate recognition of helminth products. The same chemokines that instructed intestinal repair in mice were also secreted by human macrophages, when co-cultured with immune serum and helminths. Finally, human myofibroblasts closed in vitro scratch wounds more rapidly, when stimulated with the chemokine secretions of helminth-antibody activated human macrophages. Thus, our findings reveal a novel mechanism, by which a protective antibody response can promote the repair of intestinal injury during helminth infection.

## Introduction

Infections with intestinal helminths affect more than 2 billion people globally [1,2] and drug-resistant helminths pose a considerable threat to agricultural livestock [3,4]. Helminth infections thus present a major global health concern particularly due to the propensity of these parasites to form chronic and repeated infections [5,6]. The potent immune-modulatory capacities of helminth parasites have been shaped by their long co-evolution with their hosts’ immune systems, which has resulted in a fine-tuned balance between inflammation on the one side, and parasite control on the other side [7]. In addition to their anti-inflammatory potential, helminths are increasingly recognized for their capacity to induce rapid tissue repair [8–10].

Considering the tissue migratory potential of the macroscopic larval stages, it is surprising that helminth infections are rarely associated with severe bleeding or bacterial sepsis [11]. This may partially be explained by parasite-mediated immune-modulation including the induction of IL-10 production in settings of bacterial translocation [11]. In addition, tissue dwelling nematodes typically initiate type 2 responses, which have been implicated in promoting tissue repair [12]. Helminths can trigger type 2 responses either directly [13], or through the release of alarmins (IL-25, IL-33, TSLP) or adenosine from epithelial cells following invasion [14,15].

Although the mechanisms by which helminth invasion initiates type 2 responses are increasingly well described, relatively little is known about the effector mechanisms that promote repair of host tissues following larval migration. Models of acute lung damage or liver fibrosis caused by the helminths *Nippostrongylus brasiliensis* [8,9] or *Schistosoma mansoni*, respectively have demonstrated important roles for Arginase-1 (Arg1) and insulin-like growth factor 1 (IGF-1) expressing MΦ, and IL-9R-dependent production of innate lymphoid cells (ILCs) in controlling inflammation and tissue damage [9,16,17]. However, intestinal helminth infections are often chronic and proceed without the development of excessive fibrosis, suggesting that distinct mechanisms might contribute to homeostatic wound healing in the intestine. Moreover, little is known about a potential contribution of the immune memory to these processes, yet those living in endemic regions are continuously exposed to helminths and typically suffer repeated infections.

Our previous work identified the helminth-antibody-driven activation of Arg1 expressing MΦ as an important mechanism to limit tissue disruption at early timepoints following repeated infection with the intestinal nematode *Hpb* [10]. *Hpb* is a natural parasite of mice with a strictly enteric life cycle that establishes chronic infections upon primary exposure [18], but which can be rapidly targeted by immune antibodies following repeated infections [10,19,20]. Important overlaps between mechanisms of protective immunity and wound healing during helminth infection are beginning to emerge [8], but the role of antibodies in wound healing has not been investigated.

Our previous findings suggested that the combination of immune antibodies and helminth antigens can induce the expression of several MΦ genes with potential roles in tissue repair [10]. This included the CXCR2 ligands Cxcl2 and Cxcl3, which have been implicated in cutaneous wound healing [21,22]. Here we demonstrate that intestinal wound containment was impaired in antibody- and antibody-receptor deficient mice and correlated with reduced expression of CXCR2 ligands. We further show that Fcrg-chain-signaling triggered the release of CXCL2 from MΦ and intestinal MF during helminth infection. Fcrg-dependent chemokine release by MΦ required stimulation by antibodies and helminths, whilst helminth products alone elicited the release of chemokines by MF through the dectin2 pathway, which is known to require Fcrg chain signaling. CXCR2 ligation was required for wound containment during murine helminth infection in vivo, and for efficient wound closure by human MF in vitro. Our findings reveal a dual role for helminths and immune antibodies in the trapping of invasive larvae and the promotion of tissue repair.

## Results

### Antibody- or Fcrg-deficient mice exhibit dysregulated tissue repair following challenge infection with the intestinal helminth *Hpb*

**Fig 1:**
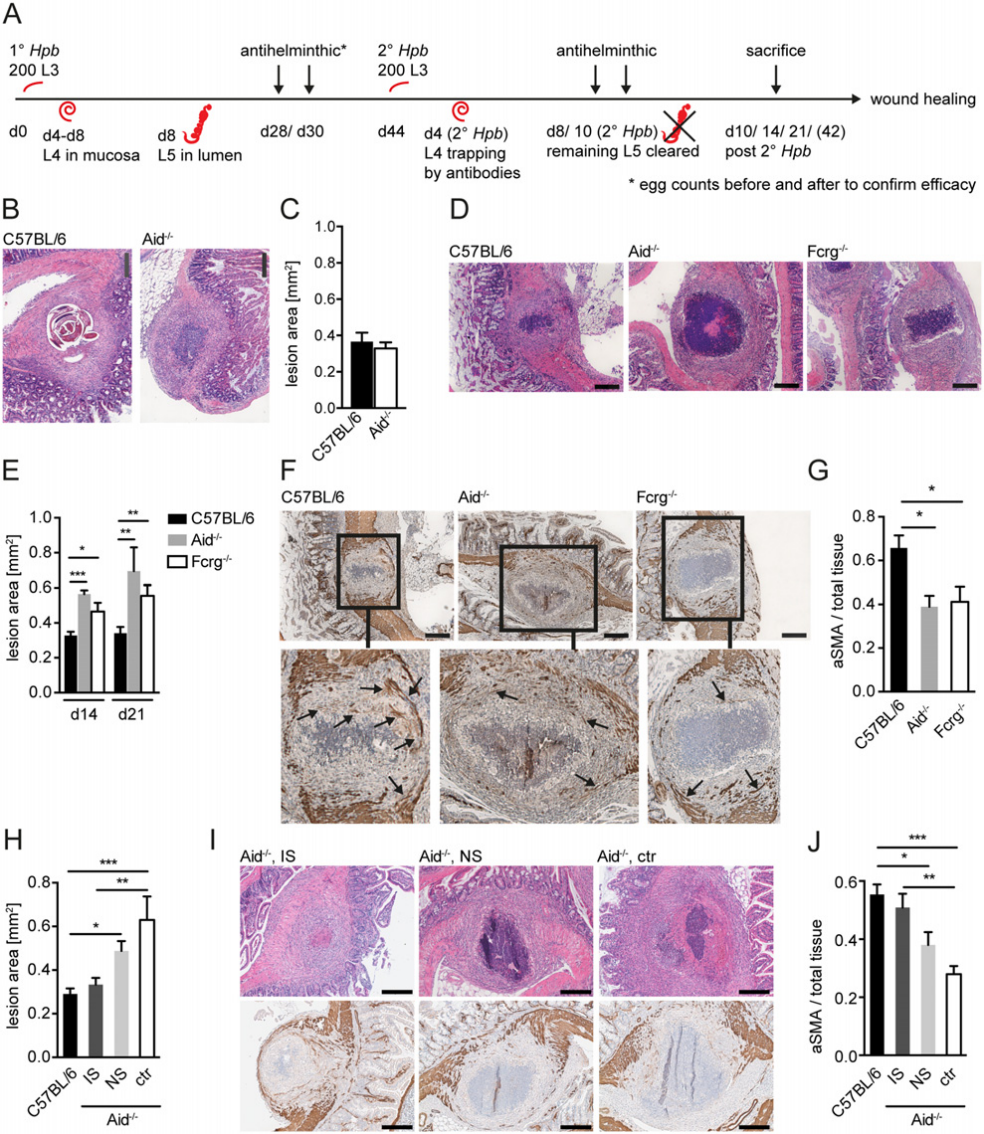
Antibody and Fcrg deficient mice display increased intestinal lesions and reduced accumulation of myofibroblasts. Mice were challenge-infected with 200 infective *Hpb* larvae (scheme of infection and antihelminthic regime depicted in (A)), small intestines were harvested at day 10, 14 or 21 p.i.; tissue sections were stained by hematoxillin and eosin (H&E) or immunohistochemistry (IHC) and wide field microscopy images were analyzed using ImageJ. (B) Representative pictures of the largest H&E-stained cross sections of lesions at day 10 p.i. in C57BL/6 or Aid^-/-^ mice; (C) Quantification of lesion area of largest cross sections (day 10 p.i.) for C57BL/6 or Aid^-/-^ mice; (D) Representative pictures of the largest H&E-stained cross sections of lesions from C57BL/6, Aid^-/-^ or Fcrg^-/-^ mice at day 14 p.i.; (E) Quantification of lesion area of largest cross sections (day 14 or 21 p.i.) for C57BL/6, Aid^-/-^ or Fcrg^-/-^ mice; (F) Representative images of IHC staining for aSMA in largest cross sections of lesions in tissues from C57BL/6, Aid^-/-^ or Fcrg^-/-^ mice; arrows indicate aSMA^+^ areas with potentially contractile morphology. (G) Quantification of aSMA-stained area in lesions of C57BL/6, Aid^-/-^ or Fcrg^-/-^ mice; (H) Quantification of lesion area of largest cross sections (day 14 p.i.) for C57BL/6 or Aid^-/-^ mice treated with immune serum from secondary infected (IS) or naïve (NS) C57BL/6 mice; (I) Representative pictures of the largest H&E-stained cross sections (top) or IHC staining for aSMA (bottom) of lesions at day 14 p.i. in C57BL/6 or Aid^-/-^ mice treated with immune serum from secondary infected (IS) or naïve (NS) C57BL/6 mice; (J) Quantification of aSMA-stained area in lesions of C57BL/6, IS, NS or untreated (ctr) Aid^-/-^ mice; All data are pooled from 2–3 independent experiments with 3–6 mice per group and presented as mean + SEM; Scale bars 200 μm.

We have previously reported an important role for antibody-mediated MΦ activation in limiting parasite-induced tissue disruption and promoting larval trapping following secondary challenge infections with *Hpb* [10]. We also noted that the same MΦ exhibited a transcriptional profile similar to the one of wound healing MΦ [10,23]. To investigate whether antibodies also impacted on tissue repair we quantified the size of intestinal lesions at various time points post secondary challenge infection (p.i.). A scheme describing the timing of *Hpb* infections, larval maturation (L3–L5) and antihelminthic treatments is shown in Fig. 1A. At day 10 p.i. we could not observe significant differences in the size of intestinal lesions between wild-type (WT) mice and mice deficient in activation induced cytidine deaminase (Aid) which fail to produce affinity-matured, class-switched antibodies [24] (Fig. 1B, C). We further observed a tendency of Aid^-/-^ mice to develop increased numbers of intestinal lesions (S1A Fig), which might be explained by the higher migratory capacity of larvae in the absence of antibodies. The cellular infiltration in the lesion underwent a dramatic change over time characterized by a shift from monocytic cells (day 4) to granulocytes (day 10) (S1B Fig). However, in agreement with our earlier findings, lesions from Aid^-/-^ and WT mice exhibited similar cellular compositions with the exception of reduced basophil numbers in the absence of Aid [10,25] (S1C-H Fig).

The finding that lesions were of similar size in Aid^-/-^ and WT mice was rather surprising as larvae had already left the intestinal mucosa of Aid^-/-^ mice at this time point, whilst they remained trapped within antibody sufficient WT lesions (Fig. 1B). We therefore additionally quantified lesion sizes in challenge-infected WT and Aid^-/-^ mice, or mice deficient in activating antibody receptors (Fcrg-chain^-/-^), at later timepoints. Intact larvae were absent from the lesions of most WT mice by day 14 p.i., possibly as a result of degradation by the large numbers of infiltrating granulocytes (Fig. 1D, S1B Fig). Interestingly, lesion size had decreased slightly but not significantly (by around 10%) by day 14–21 p.i. as compared to day 10 p.i. in WT mice whilst Aid^-/-^ and Fcrg^-/-^ mice showed significantly larger lesions (Fig. 1D, E). Impaired lesion contraction in antibody and antibody receptor deficient mice correlated with a reduced accumulation of α smooth muscle actin (aSMA) expressing cells, which include MF, the major cell type involved in wound contraction [26] (Fig. 1F, G). To further investigate the impact of antibodies on the resolution of intestinal lesions we set up experiments to investigate lesion size at even later timepoints (day 42) post secondary challenge infection. However, most of the experiments had to be halted at day 21 post infection as the majority of challenge-infected Aid^-/-^ and Fcrg^-/-^ mice showed symptoms of morbidity (most likely severe peritonitis) around this time point. Indeed only 3 Aid^-/-^ mice survived symptom free until day 42 post challenge infection. When comparing intestinal lesions in WT and these surviving Aid^-/-^ mice, an increased tendency to develop necrosis or fibrosis as well as impaired lesion resolution became apparent in the antibody deficient mice (S2A, B Fig). Increased inducible nitric oxide synthase expression in peritoneal cells in Aid^-/-^ as compared to WT mice already at day 14 post challenge infection hinted at increased peritonitis [27] in the absence of antibodies, possibly explaining the considerable morbidity in these mice late after challenge infection.

Taken together these findings suggest that antibodies act not only to trap helminth larvae but also to promote the accumulation of aSMA^+^ cells that are important for mucosal lesion contraction. In addition, the efficient (antibody-dependent) resolution of intestinal lesions might by associated with the control of peritoneal inflammation during challenge infection with intestinal helminths.

### *Hpb*-induced type 2 inflammation is intact in challenge-infected antibody deficient or FcRg deficient mice

To investigate if type 2 immunity was intact in antibody and FcRg deficient mice, we analyzed peritoneal eosinophil accumulation and type 2 cytokine (IL-4 and IL-13) production in the duodenum as key parameters of the local memory T_H_2 response. As shown in S2D Fig, large numbers of eosinophils infiltrated the peritoneal cavity during challenge infection (day 14 and 21) in WT, Aid^-/-^ and Fcrg^-/-^ mice. Moreover, IL-4 and IL-13 were both detectable in duodenal culture supernatants of all challenge-infected mice with minor reductions in Aid^-/-^ or Fcrg^-/-^ mice, respectively (S2E, F Fig). In contrast, during primary infection the levels of both cytokines were close to the levels in naïve mice (limit of detection 4 pg/ ml) (S2E, F Fig). Together these data indicate that the T_H_2 response in challenge-infected antibody and FcRg deficient mice resembles that in challenge-infected rather than in primary infected WT mice.

We reasoned the increased susceptibility of challenge-infected antibody and FcRg deficient mice to peritonitis may be due to defective lesion repair resulting in increased bacterial translocation across the intestinal epithelium. To test this hypothesis, we quantified soluble CD14 levels in the peritoneum of primary infected WT and challenge-infected WT, Aid^-/-^ or Fcrg^-/-^ mice. As shown in S2G Fig, sCD14 levels did not increase above those found in naïve mice following challenge infection of WT, Aid^-/-^ or Fcrg^-/-^ mice, whilst high levels of peritoneal sCD14 could be detected following primary infection of WT mice (day 14). These data suggest that immune memory may function to prevent bacterial translocation, but through mechanisms distinct from the impact of immune antibodies on wound contraction or peritonitis.

### Immune antibodies promote intestinal wound repair following challenge infection with *Hpb*

As our data suggested a central role for antibodies in lesion contraction during challenge *Hpb* infection, we sought to confirm a role for “immune memory” in this process. We first compared lesion sizes in primary and secondary *Hpb* infected WT mice. At day 14 and 21, primary lesions were less dense with fewer infiltrating cells (S2H Fig), but of similar size (S2I Fig) to those seen at the same time-points following challenge infection. However, it should be noted that significantly more lesions were observed in challenge-infected mice compared to primary-infected mice (S2J Fig), probably as a result of the greater type 2 immune response observed following challenge infection (S2D Fig). To determine whether lesion resolution differed between primary or challenge infected mice we next analyzed lesion size at a later time-point, day 42. By this time-point lesions had started to contract in challenge-infected, but not primary infected, mice (S2I Fig), indicating that immune memory can result in more rapid wound contraction.

To further support a role for ‘immune memory’ in lesion contraction we determined lesion size in antibody deficient (Aid^-/-^) mice receiving serum from naïve or challenge-infected WT mice. As shown in Fig. 1H, intestinal lesions in Aid^-/-^ mice that received immune serum from challenge-infected WT mice exhibited lesions of a similar size to that seen in WT mice. In contrast, transfer of naïve serum into Aid^-/-^ recipients did not result in a significant improvement of intestinal wound repair (Fig. 1H, I top panel). Moreover, the improved lesion contraction in immune serum treated Aid^-/-^ mice correlated with an increased accumulation of aSMA^+^ cells within the lesions of these mice as compared to lesions in untreated Aid^-/-^ mice (Fig. 1I bottom panel, J). These data indicate that immune memory acts to promote both lesion containment (day 14–21 post-infection) and lesion contraction (day 42 post-infection).

### Antibody-trapped helminths promote the expression of CXCL2 and CXCL3 in vivo

**Fig 2:**
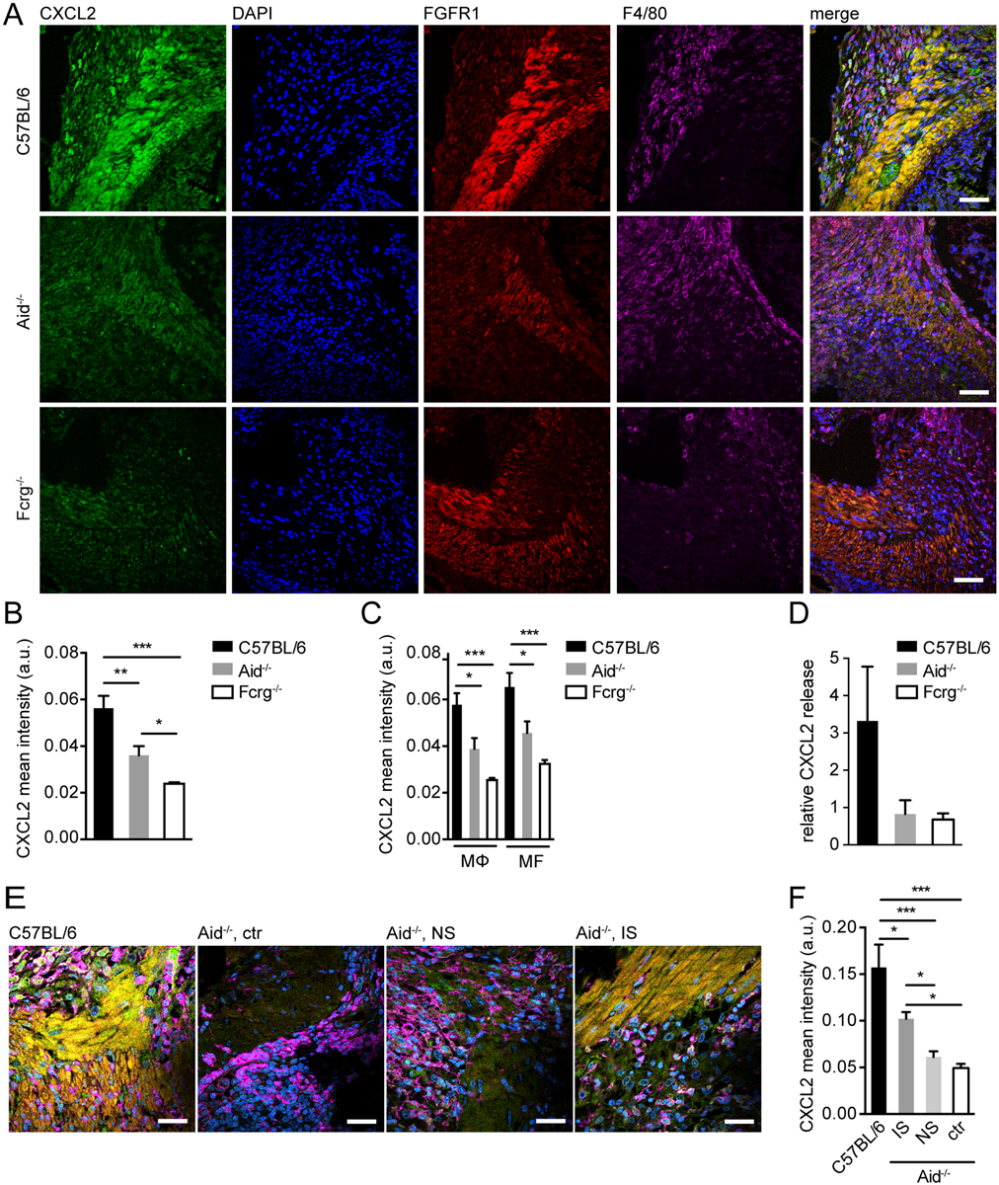
Antibody and Fcrg-dependent mechanisms trigger CXCL2 expression during helminth infection in vivo. Small intestinal tissues from *Hpb* challenge-infected mice were either used for immunofluorescence (IF) staining for CXCL2/3 (green), FGFR1 (MF, red), F4/80 (MΦ, magenta) and counterstaining by DAPI (blue) or for overnight tissue culture for ELISA quantification of CXCL2 in culture supernatants, IF quantification was performed using CellProfiler; (A) Representative images of IF staining for CXCL2, FGFR1, and F4/80 in intestinal lesions from C57BL/6, Aid^-/-^ or Fcrg^-/-^ mice; (B) Quantification of mean (“per cell”) CXCL2 fluorescence intensity in IF-stained intestinal lesions from C57BL/6, Aid^-/-^ or Fcrg^-/-^ mice; (C) Quantification of mean CXCL2 fluorescence intensity in IF-stained intestinal lesions in F4/80^high^ MΦ or FGFR1^high^ MF; (D) CXCL2 in tissue culture supernatants was quantified by ELISA and normalized to levels in naïve controls; (E) Representative images of IF staining for CXCL2, FGFR1, and F4/80 in intestinal lesions from C57BL/6 or Aid^-/-^ (control or transfer of naïve (NS) or immune serum (IS)); (F) Quantification of mean (“per cell”) CXCL2 fluorescence intensity in IF-stained intestinal lesions (treatments as in E). All data are pooled from 2–3 independent experiments with 3–6 mice per group and presented as mean + SEM; Scale bars 200 μm.

Our previous work had identified the chemokines Cxcl2 and Cxcl3 as being amongst the most highly up-regulated MΦ genes following culture of these cells together with helminth larvae and IS [10]. As CXCL2 and CXCL3 have been reported to promote repair processes, including smooth muscle cell migration [21,28], we investigated the expression of these chemokines during helminth infection in vivo. We observed that at day 14 following primary infection or day 10 following challenge infection, CXCL2 & 3 were both expressed at low levels within intestinal lesions (S3A, B Fig). However by day 14 post challenge infection, CXCL2 was abundantly expressed, particularly at the lesion edge where it co-localized with FGFR1 expressing MF and F4/80 expressing MΦ (Fig. 2A). As FGFR1 is also expressed by fibroblasts, we confirmed the co-expression of aSMA in FGFR1 expressing cells in the periphery of intestinal lesions, which identified these cells as myofibroblasts (S3C Fig). We further quantified CXCL2 expression intensity in areas with a comparable MΦ and MF infiltrate (S3D, E Fig) to exclude a potential bias due to lower numbers of MF in lesions of antibody (receptor) deficient mice. As shown in Fig. 2B, Aid^-/-^ and Fcrg^-/-^ mice presented a significantly reduced CXCL2 staining intensity on a per cell basis, which was due to lower intensities in both MΦ and MF (Fig. 2C). Interestingly, CXCL2 expression in lesions of Fcrg^-/-^ mice was significantly reduced as compared to that in Aid^-/-^ lesions, suggesting that antibody independent but FcRg chain dependent signaling events might contribute to CXCL2 production during helminth infection *in vivo*. In keeping with the observed reduction in aSMA^+^ cells in lesions of Aid^-/-^ and Fcrg^-/-^ mice (Fig. 1F, G), MF in these lesions expressed lower levels of FGFR1 (S3F Fig), which is required for MF differentiation, including the upregulation of aSMA expression [29]. As our immunofluorescence (IF) analysis could not distinguish between chemokine production or surface binding, we additionally analyzed CXCL2 secretion into supernatants from duodenal organ cultures from challenge-infected mice. Small-intestinal tissue from infected WT mice secreted 3-fold more CXCL2 as compared to naïve mice, whilst Aid^-/-^ or Fcrg^-/-^ tissues secreted levels similar to that of naïve controls (Fig. 2D). Thus, increased levels of CXCL2 might be secreted locally in response to antibody-trapped larvae. To investigate whether antibodies and antibody receptors additionally impacted on the production of the close homologue CXCL3, we performed IF stainings on the corresponding serial sections of small intestinal lesions. As shown in S3G, H Fig, CXCL3 showed a similar pattern of expression as CXCL2 with a significant reduction in Fcrg^-/-^ mice.

Finally, we analyzed CXCL2 in Aid^-/-^ mice that had received WT immune sera during challenge infection. As shown in Fig. 2E,F, transfer of immune but not naïve sera could partially restore chemokine production in antibody deficient mice. These data suggest that the CXCR2 ligands, CXCL2 & 3, are upregulated in intestinal lesions by mechanisms involving Fcrg-chain-mediated activation of MΦ and/or MF by immune antibody-trapped helminth larvae.

### Immune serum and helminth larvae trigger Fcrg-dependent CXCL2 release by MΦ in vitro

**Fig 3:**
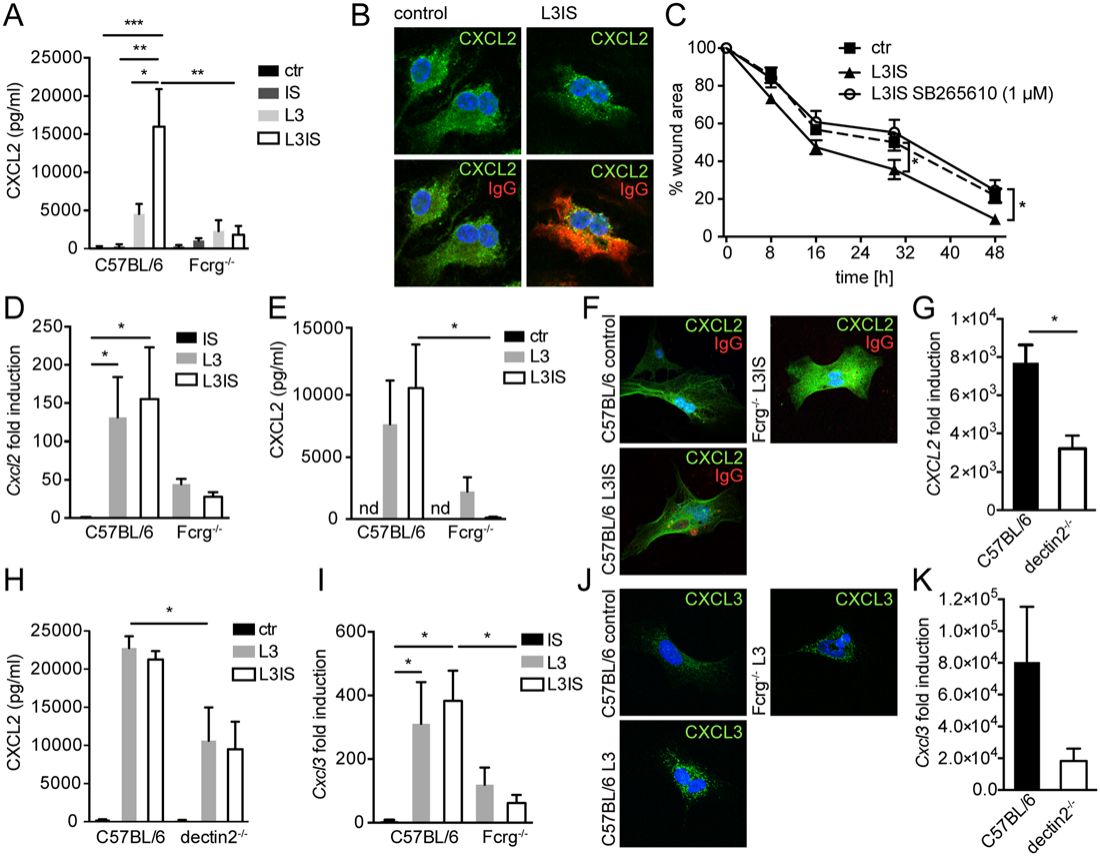
Helminths and antibodies trigger CXCL2 release by MΦ or MF via antibody-Fcrg- or Fcrg-chain/ dectin2 signaling, respectively and MF respond to MΦ-produced CXCR2 ligands in vitro. Bone marrow derived MΦ (BMM) or primary small intestinal MF from were co-cultured with *Hpb* larvae (L3) for 24h with our without immune sera (IS) from challenge *Hpb* infected mice; (A) CXCL2 in BMM culture supernatants (C57BL/6 or Fcrg^-/-^) cultured without (ctr) or with IS, L3 or both; (B) top: CXCL2 staining in control or L3IS-co-cultured BMM, bottom: IgG (red)/ CXCL2 (green) overlay; (C) Scratch wound closure by MF after addition of supernatants from BMM, cultured without (ctr) or with L3 and IS +/- CXCR2 antagonist SB265610. (D) *Cxcl2* mRNA induction in MF after IS, L3 or L3 and IS-stimulation relative to unstimulated MF; (E) CXCL2 in culture supernatants from MF, cultured without (ctr) or with L3 or L3 and IS; (F) Overlays of CXCL2 (green) and IgG (red) for unstimulated (control) MF and L3IS-co-cultured C57BL/6 or Fcrg^-/-^ MF; (G) *Cxcl2* mRNA induction in MF after L3 co-culture; (H) CXCL2 in MF supernatants after culture without (ctr) or with L3 or L3IS;(I) *Cxcl3* mRNA induction in MF after culture in the absence or presence of L3 or L3IS; (J) CXCL3 in unstimulated (control) MF or L3-co-cultured MF; (K) *Cxcl3* mRNA induction in MF after L3-co-culture; All data are pooled from at least 2 independent experiments with cells from 3–4 mice per group and presented as mean + SEM.

We had previously demonstrated that the IS-induced trapping of infective helminth larvae (”L3”) by bone marrow derived MΦ (BMM) was associated with a strong transcriptional activation of Cxcl2/3, which occurred independently of Fcrg-chain signaling [10]. To verify a direct effect of larvae and IS on CXCL2 secretion by MΦ, we quantified CXCL2 in supernatants from BMM after co-culture with helminth larvae in the presence or absence of IS. IS alone had no effect on CXCL2 release by WT BMM, whilst larvae induced CXCL2 secretion, which was enhanced by IS (Fig. 3A). Moreover, Fcrg^-/-^ BMM showed a considerably reduced CXCL2 release compared to WT BMM, when co-cultured with larvae in the presence of IS. This suggested that Fcrg-mediated activation of MΦ was responsible for the enhanced release, but not transcription of Cxcl2. Indeed, unstimulated BMM showed considerable amounts of intracellular CXCL2 (Fig. 3B), but no secretion (Fig. 3A), whilst intracellular CXCL2 levels in helminth-IS activated BMM did not reflect the strong mRNA upregulation previously observed (Fig. 3B) [10]. Thus, surface IgG receptors likely play an important role in triggering CXCL2 release by MΦ during helminth infection.

### Conditioned media from helminth-immune serum-activated MΦ drive in vitro scratch wound closure by small intestinal myofibroblasts

Crosstalk between MΦ and fibroblasts or MF is integral to the wound healing process [30]. Together, our in vitro and in vivo data suggested, that MΦ, which populate the wound early after infection [31], might be activated by antibody-trapped helminth larvae to enhance MF recruitment to intestinal lesions by secreting CXCL2/3. Hence, we tested a direct effect of MΦ-secreted CXCL2/3 by investigating scratch wound closure by MF in the presence of conditioned medium from helminth-IS activated MΦ. Addition of conditioned media from stimulated BMM significantly improved MF migration into the scratch wound area (Fig. 3C). Both CXCL2 and CXCL3 bind with high affinity to the chemokine receptor CXCR2 [32]. Thus, we studied the involvement of CXCR2 signaling in the positive effect of BMM-secreted factors on scratch wound closure by MF using the CXCR2 receptor antagonist SB265610. As shown in Fig. 3C, addition of the CXCR2 antagonist reduced the improvement of in vitro wound closure in response to conditioned media from helminth-IS activated MΦ (see also S1 Movie). These findings support a paracrine role for MΦ-secreted CXCR2 ligands in MF-mediated wound containment during helminth infection. However, once exposed to larval components, MF may also produce autocrine CXCL2/3.

### Helminth larvae induce Fcrg/ dectin2-dependent CXCL2/3 production by intestinal MF in vitro

Previous studies have shown that pro-inflammatory stimuli can elicit CXCL2 expression by stromal cells [33]. To clarify if MF represent a potential additional source of CXCL2/3 during helminth infection, we isolated small intestinal MF and performed co-cultures with helminth larvae in the absence or presence of IS. As shown in Fig. 3D, larvae potently induced Cxcl2 gene expression in MF, which in contrast to MΦ, was not augmented by the addition of IS. Moreover, MF secreted considerable levels of CXCL2 protein when co-cultured with helminth larvae (Fig. 3E), indicating that helminth larvae alone can activate MF to produce CXCL2 independently of antibodies. This was consistent with the finding that only low levels of surface IgG bound to MF cultured in the presence immune serum (Fig. 3F).

We also investigated a potential role of Fcrg-chain signaling in promoting CXCL2 production by MF. Whilst larvae alone, or larvae in combination with immune serum, lead to similar increases in CXCL2 production by WT MF, neither condition could significantly upregulate CXCL2 production by Fcrg^-/-^ MF (Fig. 3E). This suggested that another γ-chain dependent (antibody independent) event was required for the upregulation of MF CXCL2 production by helminth larvae. In addition to surface Fcr-mediated recognition of antibody-coated helminths, helminthic glycan structures can be recognized via pathogen recognition receptors such as dectin1 or 2, the mannose receptor (MR) or DC-SIGN [34,35]. Amongst these receptors, dectin2 is unique in its requirement of the Fcrg-chain for signaling [36]. Thus, we studied a potential involvement of dectin2 in the helminth-driven induction of CXCL2 production by intestinal MF. As shown in Fig. 3G, H dectin2^-/-^ MF showed a significant reduction in helminth-induced Cxcl2 mRNA expression and protein release, suggesting that dectin2 recognition of larval components contributes to the upregulation of chemokine production by MF. Interestingly a more dramatic defect was observed for Fcrg^-/-^ as compared to dectin2^-/-^ MF, which may indicate that additional Fcrg-chain coupling C type lectins are involved [37]. Finally, the pattern of expression of CXCL3 in wildtype and Fcrg^-/-^ and dectin2^-/-^ MF closely paralleled the one of CXCL2 (Fig. 3I-K).

### Inhibition of CXCR2 signaling leads to delayed MF accumulation and increased lesion size following helminth infection

**Fig 4:**
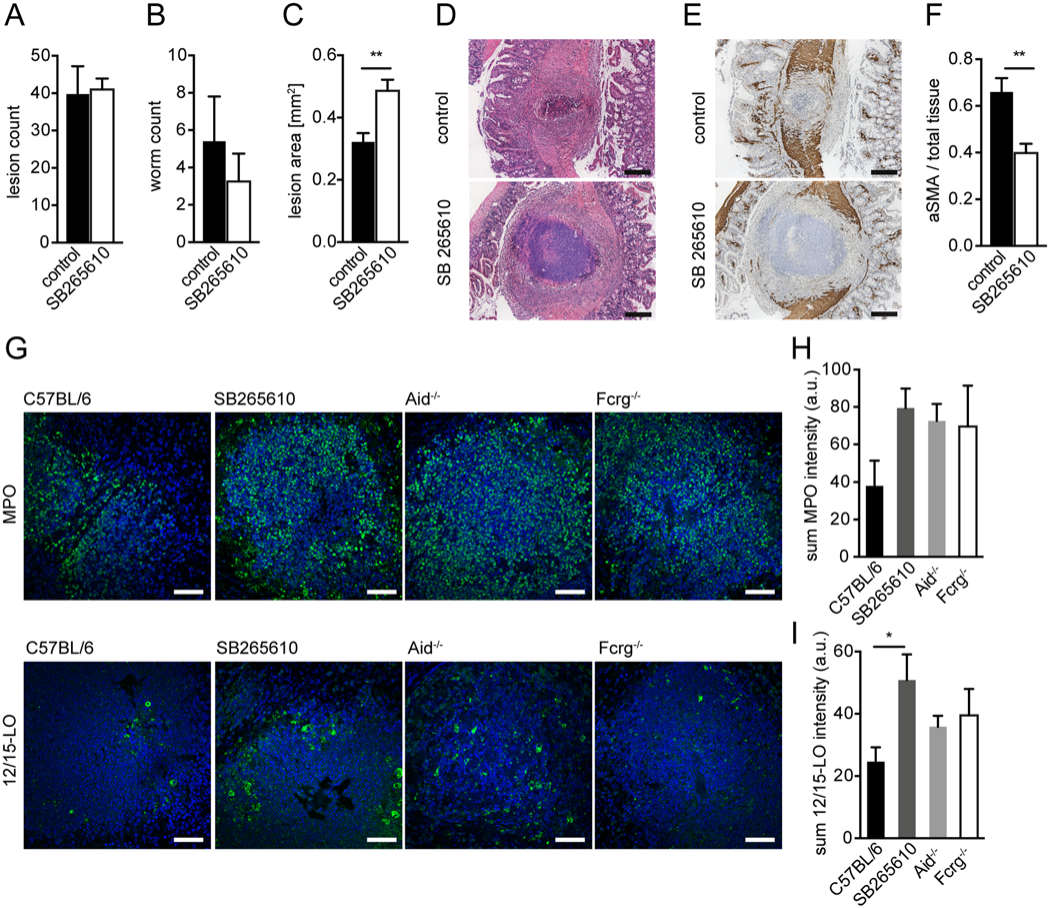
Inhibition of CXCR2 signaling leads to delayed MF accumulation and increased lesion size without affecting granulocyte recruitment in vivo. C57BL/6 mice were treated or not with the CXCR2 antagonist SB265610 (3 mg/kg per oral gavage, once daily) during challenge infection with *Hpb*; small intestines were harvested for parasitology and histology at day 14 p.i. (A) Lesions were counted in small intestines of untreated (control) and SB265610 treated mice; (B) Adult worms were counted in opened small intestines of untreated (control) and SB265610 treated mice; (C) The area of the largest lesion cross sections was quantified in H&E-stained tissue sections from untreated (control) and SB265610 treated mice; (D) Representative H&E-stained cross sections of intestinal lesions in untreated (control) and SB265610 treated mice; Scale bars 200 μm. (E) Representative IHC-staining for aSMA in lesions of untreated (control) and SB265610 treated mice; Scale bars 200 μm. (F) Quantification of aSMA-stained area in lesions of untreated (control) and SB265610 treated mice was performed using ImageJ; (G) Small intestinal tissue sections from challenge-infected untreated C57BL/6 (control) and SB265610 treated C57BL/6 mice or Aid^-/-^ or Fcrg^-/-^ mice were IF-stained for the neutrophil marker MPO (upper panel, green) or the eosinophil marker 12/15-lipoxygenase (12/15LO) (lower panel, green), followed by counterstaining with DAPI (blue); Scale bars 50 μm. (H) Total Fluorescence intensity of MPO was quantified using CellProfiler; (I) Total Fluorescence intensity of 12/15LO was quantified using CellProfiler; All data are pooled from at least 2 independent experiments with 3–6 mice per group and presented as mean + SEM.

Our earlier data suggested increased expression of CXCR2 ligands in helminth-induced lesions in vivo and a role for CXCR2 in MF migration in response to MΦ-secreted chemokines in vitro. To assess a potential role for CXCR2 in intestinal repair during helminth infection in vivo, we treated mice by the orally active CXCR2 antagonist SB265610 [22]. As CXCR2-driven neutrophil recruitment has been reported to contribute to protection against the helminth *Strongyloides stercoralis* [38], we first enumerated intestinal lesions and luminal parasites at day 14 post *Hpb* challenge infection. No significant effect of CXCR2 antagonist treatment was noted for either lesion or parasite numbers (Fig. 4A, B). We next compared the size of intestinal lesions between vehicle and SB265610 treated mice at day 14 post challenge infection. As shown in Fig. 4C, D, inhibition of CXCR2 signaling resulted in the development of larger and more irregular intestinal lesions. When analyzing intestinal aSMA expression, we observed a striking defect in the accumulation of aSMA^+^ cells within intestinal lesions of SB265610 treated mice (Fig. 4E, F), suggesting that CXCR2-ligands might also contribute to MF accumulation and wound containment in vivo. This phenotype observed in SB265610 treated mice closely paralleled the phenotype of Aid^-/-^ and Fcrg^-/-^ mice suggesting that defective CXCL2/3 expression was responsible for impaired wound healing in the absence of immune antibodies or Fcrg chain signaling.

### CXCR2-signaling is not required for collagen deposition following helminth infection

In addition to their contractile function, MF promote wound healing by depositing extracellular matrix components, including collagen. Thus, we investigated whether the reduced accumulation of MF associated with CXCR2 blockade also impacted on collagen deposition within intestinal lesions. Mice treated with the CXCR2 antagonist showed normal collagen levels following helminth infection (S4A, B Fig) suggesting that this pathway is not involved in collagen deposition. However antibody (receptor) deficient mice displayed increased lesional collagen levels (S4C, D Fig). This may suggest that collagen production on a per cell basis is increased in Aid^-/-^ and Fcrg^-/-^ mice, which could be explained by the potential of trapped larvae to suppress collagen expression, whilst inducing collagen-remodeling matrix metalloproteinase and anti-fibrotic cyclooxygenase-2 (Cox2) enzymes in intestinal MF (S4 E-I Fig).

### CXCR2 signaling is dispensable for granulocyte recruitment to intestinal lesions

Several studies have implicated CXCR2 in granulocyte recruitment during helminth infection [38,39]. We therefore determined the number of neutrophils and eosinophils in lesions of SB265610 treated mice and in Aid^-/-^ or Fcrg^-/-^ mice. We observed considerable levels of neutrophil myeloperoxidase (MPO), a widely used neutrophil marker, in the center of WT lesions at day 14 p.i. (Fig. 4G). However, when comparing MPO expression between WT, SB265610-treated or antibody (receptor) deficient mice, we observed an increase rather than a decrease in neutrophils (Fig. 4G upper panel, H), indicating that CXCR2 ligands are not required for neutrophil recruitment to intestinal lesions during challenge infection.

As neutrophils can contribute to tissue damage during helminth infection [9,40], we attempted to deplete neutrophils in the small intestine of Fcrg^-/-^ mice to test a potential contribution of the increased neutrophil infiltrate to the impaired repair observed in these. Whilst neutrophils were successfully depleted in the peripheral blood of challenge infected Fcrg^-/-^ mice (S5A Fig), high numbers of MPO expressing cells were still observed in intestinal lesions of mice that had been treated with a neutrophil-depleting anti-Ly6G antibody (S5B Fig). However, we could not observe a clear correlation between the quantity of MPO staining within intestinal lesions and lesion size (S5C Fig), suggesting that the increased neutrophil accumulation in antibody deficient mice does not play a major role in their reduced capacity to repair helminth-induced lesions.

Using 12/15-lipoxygenase (12/15LO) as a marker for eosinophils (see S1 Text, S5D Fig), we observed high numbers of eosinophils, many of which appeared de-granulated, within lesions of all mice (Fig. 4G lower panel, I). Similarly to neutrophils, mice treated with a CXCR2 antagonist showed increased eosinophil numbers. Taken together, these findings implicate CXCR2 as an important immune-modulating pathway, which functions to promote wound contraction following helminth infection but does not contribute to granulocyte recruitment.

### Conditioned media from helminth-immune serum activated human MΦ promotes in vitro scratch wound closure by human MF in a CXCR2 dependent fashion

**Fig 5:**
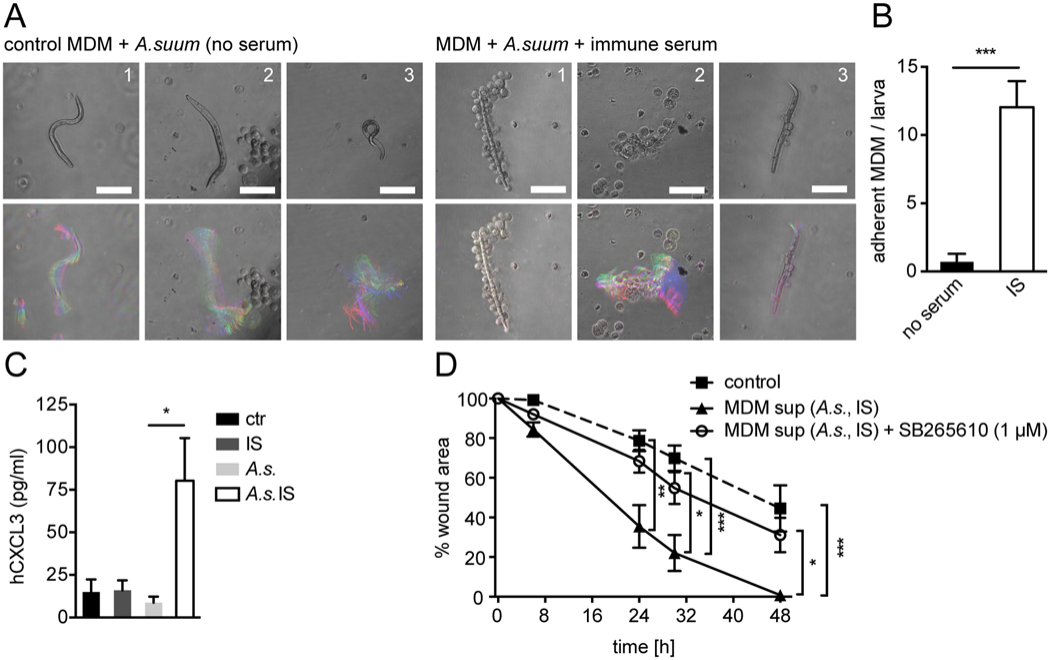
Conditioned media from *A*. *suum*-immune serum-activated MΦ promote in vitro scratch wound closure by human MF in a CXCR2 dependent fashion. (A) Human MDM from 3 healthy blood donors (1–3) were co-cultured with *A*. *suum* larvae in the absence or presence of IS from challenge-*A*.*suum* infected pigs and time-lapse movies were recorded (2 frames/ s, 1 min); upper panel: representative snap shots at T = 0; lower panel: representative temporal color code images (120 frames, 1 min)—colors represent different time points; Scale bars 100 μm; (B) Adherent MDM per *A*. *suum* larva were counted in time-lapse movies; (C) CXCL3 in culture supernatants from human MDM cultured with or without immune sera from *Ascaris*-infected pigs (IS), *Ascaris suum* larvae (*A*.*s*.) or both (*A*.*s*. IS) quantified by ELISA; (D) Scratch wound closure by human MF cultured in the presence of conditioned media from MDM (MDM sup) stimulated with *A*.*s*. IS +/- the CXCR2 antagonist SB265610; All data are pooled from at least 3 independent experiments with n = 3 co-cultures in each and presented as mean +/-SEM.

To determine whether helminth- and antibody-induced CXCL2/3 production plays a role in wound contraction in response to other helminth species we performed co-cultures of *Ascaris suum* (*A*. *suum*) larvae and IS from challenge-*A*. *suum*- infected pigs with human monocyte-derived MΦ (MDM). The pig parasite *A*. *suum* and the human parasite *A*. *lumbricoides* are closely related and cross-infections are common in endemic areas [41]. When incubated with IS, MDM adhered to *A*. *suum* larvae (Fig. 5A upper panel, B), which was associated with partial immobilization of infective larvae (Fig. 5A lower panel). This finding was in keeping with old literature, demonstrating the interaction of MΦ and *A*. *suum* after stimulation with IS [42].

Upon stimulation with phorbol esters and calcium ionophores or LPS, human and pig monocytes or MΦ can produce CXCL3, which is homologue to mouse CXCL2 and which binds to CXCR2 with high affinity [43,44]. Thus, we quantified CXCL3 production by human MDM after co-culture with *A*. *suum* larvae in the absence or presence of IS by ELISA. As shown in Fig. 5C MDM which had been co-cultured with *A*. *suum* larvae and IS secreted significantly larger amounts of CXCL3 as compared to culture with larvae alone. Next, we performed an in vitro wound-healing assay using primary human fibroblasts, which had been differentiated into MF by culture in the presence of TGFβ1 for 48h. Human MF (hMF) cultured in the presence of conditioned media from unstimulated MDM exhibited a reduction in the wound area by around 50% after 48h following scratching (Fig. 5D). In contrast, when conditioned media from *A*. *suum-*IS-activated MDM were added, hMF closed the scratch wound significantly faster, reaching complete wound closure after 48h (Fig. 5D, S2 Movie). We also tested if *A*. *suum* secreted products directly impacted on scratch wound closure by hMF. However, addition of *A*. *suum* products to hMF monolayers after scratch wounding had no significant effect on wound closure (S5E Fig), suggesting that mainly MΦ-secreted factors were responsible for accelerated scratch closure in the presence of conditioned media. Moreover, addition of the CXCR2 antagonist SB265610 abrogated the positive effect of *A*.*suum*-IS-activated MDM supernatants on in vitro wound closure by hMF (Fig. 5D, S2 Movie), suggesting that CXCR2 signaling is largely responsible for this effect.

Taken together these findings suggest that helminth-antibody-MΦ interactions can promote CXCR2 dependent wound closure during helminth infection across distinct host and parasite species.

## Discussion

The capacity of helminths to induce rapid and efficient tissue repair has long been recognized, but effector mechanisms responsible for wound healing have only recently begun to be determined. Here we demonstrate that antibodies, which contribute to protective immunity against several helminth species [19,45,46], additionally contribute to wound contraction. Helminths damage host tissues not only by breaching physical barriers but also by driving strong inflammatory responses including the recruitment of tissue-destructive granulocytes [9,47]. Inflammatory cell recruitment is particularly large and rapid during memory type 2 responses, when tissue migrating larvae are also trapped and killed [31,48]. Our study reveals a novel mechanism by which the protective antibody response also participates in wound healing. Thus, when mounting a protective memory response, the mammalian host uses parallel mechanisms to ensure the limitation of infection and the repair of tissue damage at the same time.

Of note, delayed type 2 inflammation during primary helminth infection was associated with higher soluble CD14 levels in the peritoneal cavity, thus implicating the rapid and strong inflammatory response during challenge infection in the control of bacterial translocation (in the face of helminth triggered tissue damage). However, the striking morbidity of antibody and FcRg deficient mice at late time points after challenge infection suggested that this strong inflammatory response can (potentially) be harmful if not properly controlled or resolved. Thus, helminth products and antibody-FcRg signaling possibly contribute to wound healing on several levels by promoting lesion containment and contraction whilst also limiting excessive peritoneal inflammation.

Although we only detected low levels of CXCL2/3 following primary helminth infection, our study does not rule out a role for antibodies or CXCL2/3 in promoting tissue repair in the absence of immunological memory. The identification of dectin-2 as an innate signaling event mediating the induction of CXCL2/3 production by helminth products may suggest that these chemokines can indeed be produced even in the absence of antibodies. The finding that FcRg deficient mice showed a stronger reduction in chemokine levels during helminth infection further supports a role for dectin-2/ FcRg chain triggered CXCL2/3. However the similar wound healing phenotype in antibody and FcRg deficient mice suggests that timely, CXCR2-driven lesion contraction during challenge infection may require the concerted activation of MΦ and MF by both antibodies and helminth products. Previous studies have identified several innate immune mechanisms that contribute to the repair of helminth-induced injury following primary infection [9,17,49,50]. Here, we observed that lesions in primary infected mice were of similar size as compared to those in secondary challenge-infected mice at day 14 and 21 post infection, which might be explained by the delayed inflammatory response during primary infection. However, primary lesions failed to contract to the same extent as secondary lesions by day 42 post infection, thus supporting a role for adaptive T_H_2 immune memory in intestinal wound repair. Moreover, the transfer of immune serum from challenge *Hpb* infected, but not from naïve, WT mice into challenge-infected Aid^-/-^ recipients could rescue the wound healing defect in these mice. This finding supports the hypothesis that immune antibodies play a major role in the containment and contraction of intestinal lesions following helminth infection. Whether the wound healing-promoting effect of immune serum mainly depends on specificity towards the worm or on different Fc functionality (e.g. through isotype switching or glycosylation) is an open question, which should be addressed in future studies.

Taken together, our work suggests that immune memory activates particularly potent and timely repair responses. It also represents the novel identification of an adaptive immune mechanism, antibody production, that can promote wound healing following helminth infection [8]. Two studies, including our own work, showed that antibody-activated Arg1 expressing MΦ limit the motility of tissue-dwelling helminth larvae, thereby preventing tissue damage during early challenge infection with *Hpb* or *N*. *brasiliensis*, respectively [10,48,51]. Our current study now demonstrates that antibody-trapped helminths modulate gene expression in MΦ and intestinal MF to improve wound contraction also at later stages of helminth infection.

Reduced peripheral MF in Aid^-/-^ and Fcrg^-/-^ mice were associated with increased lesion size and enhanced granulocyte infiltration at late timepoints post-infection, which could be reproduced by CXCR2 blockade. In contrast to the importance of CXCR2 for neutrophil recruitment to other tissues [21,39,52], CXCL2/3 thus appear to play redundant roles in neutrophil recruitment during enteric nematode infection. Instead, other chemoattractants such as complement component C5a, leukotriene B_4_ or platelet activating factor (PAF) may recruit neutrophils to intestinal lesions [53]. The increased granulocyte infiltrate in CXCR2-inhibited or Aid^-/-^ and Fcrg^-/-^ mice might further suggest that MF, which are recruited to helminth-induced intestinal lesions, can exert an anti-inflammatory function by limiting granulocyte accumulation. Interestingly, intestinal MF cultured with helminth larvae upregulated the expression of cyclooxygenase-2, which serves anti-fibrotic and anti-inflammatory functions in the gut, including limiting neutrophilic inflammation [54,55]. Even if we did not observe a correlation between the extent of neutrophil infiltration and lesion size, our study does not rule out the possibility that the increased granulocyte accumulation in the lesions of antibody and Fcrg deficient or CXCR2-inhibited mice contributes to the impaired repair response in these mice. Future studies could thus address how helminths may modulate MF and granulocyte function to prevent excessive inflammation and instruct homeostatic wound healing.

Our study describes a previously unrecognized function of helminth-induced CXCR2-ligands in intestinal wound containment, thus implicating the CXCL2/3-CXCR2 chemokine axis in repair of mucosal tissues. Whilst little was known about a potential involvement of CXCL2/3 in intestinal repair, the role of CXCR2 signaling in cutaneous wound healing after chemical damage or incision wounding is well-described [21,22]. In such settings of sterile damage, CXCR2 contributed to several of the central processes of skin repair, including keratinocyte migration, re-epithelialization and neovascularization. Moreover, CXCR2 was involved in the IL-33-induced repair of *Staphylococcus aureus*-infected wounds [56]. Our findings build on this work by additionally demonstrating that CXCR2 ligands act on MF to promote wound healing in the small intestine. Thus, it is likely that CXCR2 signaling is a conserved repair mechanism involved in the restoration of barrier integrity at different body surfaces in the absence or presence of infectious agents. Of note, CXCR2 can bind other ligands (CXCL1, CXCL5, CXCL7 and CXCL8/ IL-8) in addition to CXCL2 and CXCL3. Although these chemokines were not induced in macrophages in response to helminth larvae and immune serum [10], our data does not rule out a contribution of CXCR2 ligands other than CXCL2/3 to lesion contraction during helminth infection *in vivo*. It should also be noted that we cannot entirely exclude an impact of antibody-FcRγ signaling on the local secretion of type 2 cytokines, e.g. by basophils [57], which might additionally contribute to intestinal wound repair during secondary helminth infection.

Our findings have important clinical implications as CXCR2 has been suggested as a drug target for inflammatory diseases [58]. Together with other studies demonstrating crucial roles for the CXCL2/3-CXCR2 axis in repair and homeostasis [21,22,59], our findings argue for a careful evaluation of the therapeutic inhibition of this pathway. Future studies could address the potential of CXCR2 agonists and/or antibody-helminth-preparations to improve repair in settings of impaired wound healing responses (e.g. diabetic ulcers) or after surgery.

## Methods

### Ethics statement

All mouse experiments were approved by the office Affaires vétérinaires (1066 Epalinges, Canton Vaud, Switzerland) with the authorization number 2238 according to the guidelines set by the service de la consummation et des affaires vétérinaires federal (Canton Vaud, Switzerland). Mice were euthanized using carbon dioxide. All procedures involving pigs were performed in strict accordance with the recommendations in the Guide for the Care and Use of Laboratory Animals of the National Institutes of Health after review and approval by the Beltsville Area Animal Care and Use Committee under protocol number 10–012.

### Mice

Mice (strains see S1 Text) were bred and maintained under specific pathogen free conditions at the École Polytechnique Fédérale (EPFL) de Lausanne, Switzerland.

### Infection and parasitology

Mice were infected with 200 *H*. *polygyrus bakeri* larvae and two courses of antihelminthic cobantril were administered at days 28 and 30 p.i. Mice were re-infected with 200 larvae at day 44 and two courses of cobantril were administered at days 52 and 54 p.i. in some experiments. The CXCR2 antagonist SB265610 [22,60] (Tocris Bioscience, Bristol, UK) was administered (3 mg/ kg) once daily from day 0 p.i. by oral gavage. Mice were sacrificed at day 4–42 post challenge infection. *Ascaris suum* larvae were obtained from infective eggs according to published procedures [61]. See also S1 Text.

### Pig sera

*A*. *suum*-challenge infected pigs (see also S1 Text) were bled at day 14 p.i. for serum collection.

### In vitro cultures of MΦ and MF

Murine bone marrow derived MΦ (BMM) or human MDM were prepared as described previously [10,62]. Mouse small intestinal MF were obtained by culturing lamina propria cells isolated from the small intestine of naïve C57BL/6 mice according to previously published methods [63,64]. Human primary gingival fibroblasts were differentiated into MF by culture with TGFβ1 for 48h. See also S1 Text.

### Histology

Serial paraffin sections of small intestines (“Swiss rolls”) were stained with hematoxylin and eosin or Sirius red. Lesions were identified by light microscopy and serial sections were used for immune staining and imaging (see also S1 Text).

### Flow cytometry

Cells isolated from lesions were stained with fluorescently labeled monoclonal antibodies (see S1 Text) and acquired on a BD LSRII flow cytometer (BD, Franklin Lakes, NJ).

### qPCR

RNA extraction and qPCR analysis was performed as described elsewhere [10] (for qPCR primer sequences see Table S1 in S1 Text).

### ELISA

Concentrations of mouse CXCL2, IL-4 or IL-13 in cell or intestinal (upper duodenum) culture supernatants were quantified by using an anti-mouse CXCL2 ELISA kit (Sigma Aldrich, Buchs, Switzerland) or eBbioscience Ready-SET-Go! IL-4 or IL-13 kits (eBioscience, San Diego, CA). Human CXCL3 was quantified using an anti-human CXCL3 ELISA Kit (LuBioScience, Luzern, Switzerland).

### In vitro wound healing assay

A scratch was introduced into MF monolayers using a 200-μl pipette tip according to published methods [65] and images were recorded using an Olympus Cell’R system with a UPLAN FL 10x phase objective. The scratch area was quantified using ImageJ and normalized to the area in the same position at T = 0.

### Statistics

Mann-Whitney test or one-way ANOVA followed by Holm-Sidak’s multiple comparisons test was used to compare the means of two groups or multiple groups, respectively.

## Supporting Information

S1 Fig Antibody deficient mice show normal local inflammatory cell recruitment during early challenge infection. Lesions in *Hpb* challenge-infected C57BL/6 or Aid^-/-^ mice were counted or dissected for cell isolation followed by flow cytometric analysis. Gating was performed as published previously [10]. (A) Intestinal lesion count at day 10 post *Hpb* challenge infection; (B) Representative forward side scatter plots for cells isolated from intestinal lesions of challenge-infected mice at day 4 (left) or day 10 (right); (C-H) Flow cytometric analysis of cell populations at day 4 or 10 of challenge infection in lesions from C57BL/6 or Aid^-/-^ mice: (C) CD206^high^ macrophages, (D) CD206^intermediate^ macrophages, (E) Neutrophils, (F) Ly6C^+^ monocytes, (G) Eosinophils, (H) Basophils. Data are pooled from 2 independent experiments with 4–6 mice per group and presented as mean + SEM.
(TIF)

S2 Fig Challenge infected AID^-/-^ and primary infected WT mice show impaired lesion resolution at day 42 post *Hpb* infection; T_H_2 immunity and control of bacterial translocation are intact in challenge-infected Aid^-/-^ and Fcrg^-/-^ mice, but reduced during primary infection. (A) Masson Trichrome staining (light pink: cytoplasm, dark pink: muscle/ keratin or necrotic tissue, blue: collagen) of intestinal lesions in challenge infected C57BL/6 WT mice or Aid^-/-^ at day 42 post infection; (B) Quantification of lesion area of largest cross sections (day 42 p.i.) for C57BL/6 or Aid^-/-^ mice (n = 3); (C) Expression of iNOS mRNA relative to GAPDH in total peritoneal wash cells (day 14 p.i.); (D) Number of eosinophils in the peritoneal wash from naïve, primary (1°) or secondary challenge-infected (2°) WT, Aid^-/-^ or FcRg^-/-^ (day 14 post infection); (E-F) Quantification of IL-4 (E) or IL-13 (F) in intestinal culture supernatants from naïve (dashed line), primary or challenge-infected WT, Aid^-/-^ or FcRg^-/-^ mice (day 14 post infection); (G) Quantification of soluble CD14 levels in the peritoneal wash from naïve (dashed line), primary (1°) or secondary challenge-infected (2°) WT, Aid^-/-^ or FcRg^-/-^ (day 14 post infection); (H) Upper panel: Representative H&E images of the centre of intestinal lesions in primary and secondary *Hpb* infected mice (day 14), Lower panel: Representative H&E images of lesions at day 14, 21 and 42 of primary infection (I) Quantification of lesion area during primary and secondary infection over time; (J) Enumeration of intestinal lesions (visible by eye) in the small intestine of primary or secondary *Hpb* infected WT mice; Data are pooled from 2 independent experiments with 3–6 mice per group and presented as mean + SEM.
(TIF)

S3 Fig Expression of CXCL2 and CXCL3 during primary infection and early challenge infection. Duodenal tissues from primary or secondary *Hp*b infected mice were IF-stained for CXCL2 or CXCL3 together with FGFR1 and F4/80 followed by counterstaining with DAPI. (A) Upper panel: CXCL2/ FGFR1/ F4/80 staining at day 10 post challenge infection, lower panel: CXCL2/ FGFR1/ F4/80 staining at day 14 post primary infection; (B) Upper panel: CXCL3/ FGFR1/ F4/80 staining at day 10 post challenge infection, lower panel: CXCL3/ FGFR1/ F4/80 staining at day 14 post primary infection; (C) FGFR1 and aSMA staining at day 14 post challenge infection; (D) Total F4/80 intensities for the corresponding regions used for the CXCL2 quantification in Fig. 2B (see main text). (E) Total FGFR1 intensities for the corresponding regions used for the CXCL2 quantification in Fig. 2B (main text). (F) Mean FGFR1 intensities for the corresponding regions used for the CXCL2 quantification in Fig. 2B (main text). Data are pooled from 2 independent experiments with 5 mice per group and presented as mean + SEM. (G) Representative images of IF staining for CXCL3 (upper panel) or overlays of CXCL3 (green), FGFR1 (red), F4/80 (magenta) and DAPI (blue) (lower panel) in intestinal lesions from C57BL/6, Aid^-/-^ or Fcrg^-/-^ mice; (H) Quantification of mean CXCL3 fluorescence intensity in IF-stained intestinal lesions from C57BL/6, Aid^-/-^ or Fcrg^-/-^ mice; All data are pooled from 2–3 independent experiments with 3–6 mice per group and presented as mean + SEM.
(TIF)

S4 Fig Antibody deficiency, but not CXCR2 blockade impacts on collagen deposition in intestinal lesions of *Hpb* infected mice; larvae modify remodeling genes by intestinal MF in vitro. (A-D) Intestinal tissues from challenge-infected C57BL/6, Aid^-/-^ or Fcrg^-/-^ mice were stained for collagen with Sirius red and Sirius red staining in intestinal lesions was quantified using ImageJ; (E-I) Expression of remodeling genes in C57BL6 or Fcrg^-/-^ MF after co-culture with IS, larvae (L3) or both (L3IS) was analyzed by qPCR and normalized to expression levels in unstimulated MF; (A) Quantification of collagen staining within intestinal lesions of untreated control mice and mice treated with the CXCR2 antagonist SB265610; (B) Representative images of Sirius red-stained lesions of control and SB265610-treated mice; (C) Quantification of collagen staining within intestinal lesions of C57BL/6, Aid^-/-^ or Fcrg^-/-^ mice; (D) Representative images of Sirius red-stained lesions of C57BL/6, Aid^-/-^ or Fcrg^-/-^ mice; (E) Fold induction of Collagen 1 A1 (*Col1a1*) mRNA; (F) Collagen 3 A1 (*Col3a1*) mRNA; (G) fold induction of matrix metalloproteinase 9 (*Mmp9*) mRNA; (H) fold induction of *Mmp10*, (I) fold induction of cyclooxygenase 2 (*Cox2*) mRNA in in vitro cultured intestinal MF. Data are pooled from 2 independent experiments with tissues or cells from 3–6 mice per group and presented as mean + SEM.
(TIF)

S5 Fig Accumulation of MPO^+^ cells does not correlate with lesion size; 12/15-lipoxygenase marks eosinophils accumulating in intestinal lesions during primary and challenge helminth infection. Fcrg^-/-^ mice were treated with an anti-Ly6G antibody (1mg/day, i.p.) between day 10 and 14 of challenge infection to deplete neutrophils. (A) Neutrophil depletion in the peripheral blood of challenge-infected Fcrg^-/-^ mice (day 14 p.i.), left: isotype control (ITC), right: anti-Ly6G (B) Small intestinal tissue sections from challenge-infected Fcrg^-/-^ mice (ITC or Ly6G treated) were IHC-stained for MPO. (C) The size of individual lesions (in challenge-infected WT, Fcrg^-/-^ ITC/ Ly6G, Aid^-/-^ ctr/ NS/ IS) was plotted against the area of MPO^+^ cells within each lesion. (D) Representative images of IHC staining for 12/15-lipoxygenase (12/15LO) in intestinal lesions of *Hpb* infected BalbC or eosinophil deficient dblGata1 mice; upper panel: 12/15LO staining at day 14 of primary infection; lower panel: 12/15LO staining at day 14 of challenge infection. (E) Scratch wound closure by human myofibroblasts in the absence or presence of *Ascaris suum* (A.s.) products; Representative data from 2 independent experiments are shown.
(TIF)

S6 Fig Suggested mechanism of the helminth product- and antibody-driven macrophage-myofibroblast crosstalk leading to intestinal wound repair during challenge infection with the helminth *Heligmosomoides polygyrus bakeri* (Hpb). (TIF)

S1 Movie Conditioned media from BMM after co-culture in the presence of *Hpb* larvae and immune serum accelerate in vitro scratch wound closure by intestinal MF in a CXCR2 dependent fashion. Representative images at T = 0, 30 and 48h after scratch wounding of primary mouse intestinal MF monolayers and after addition of medium from control (unstimulated BMM) or L3IS-stimulated BMM in the presence or absence of the CXCR2 antagonist SB265610 (1 μM).
(MP4)

S2 Movie Conditioned media from human MDM after co-culture in the presence of *A*. *suum* larvae and immune serum promote in vitro scratch wound closure by primary human MF *via* CXCR2. Representative images at T = 0, 30 and 48h after scratch wounding of primary human gingival MF monolayers and after addition of medium from control (unstimulated MDM) or *A*.*suum*-IS-stimulated MDM in the presence or absence of the CXCR2 antagonist SB265610 (1 μM).
(MP4)

S1 Text Supporting information. Detailed Materials and Methods including antibodies and primer sequences and description of 12/15-lipoxygenase as a marker for intestinal eosinophils.
(DOCX)
