# Supplementary figures and images for: Immune Antibodies and Helminth Products Drive CXCR2-Dependent Macrophage-Myofibroblast Crosstalk to Promote Intestinal Repair
Source: PLoS Pathog. 2015 Mar 25;11(3):e1004778. doi: 10.1371/journal.ppat.1004778 (PMC4373753; doi:10.1371/journal.ppat.1004778)

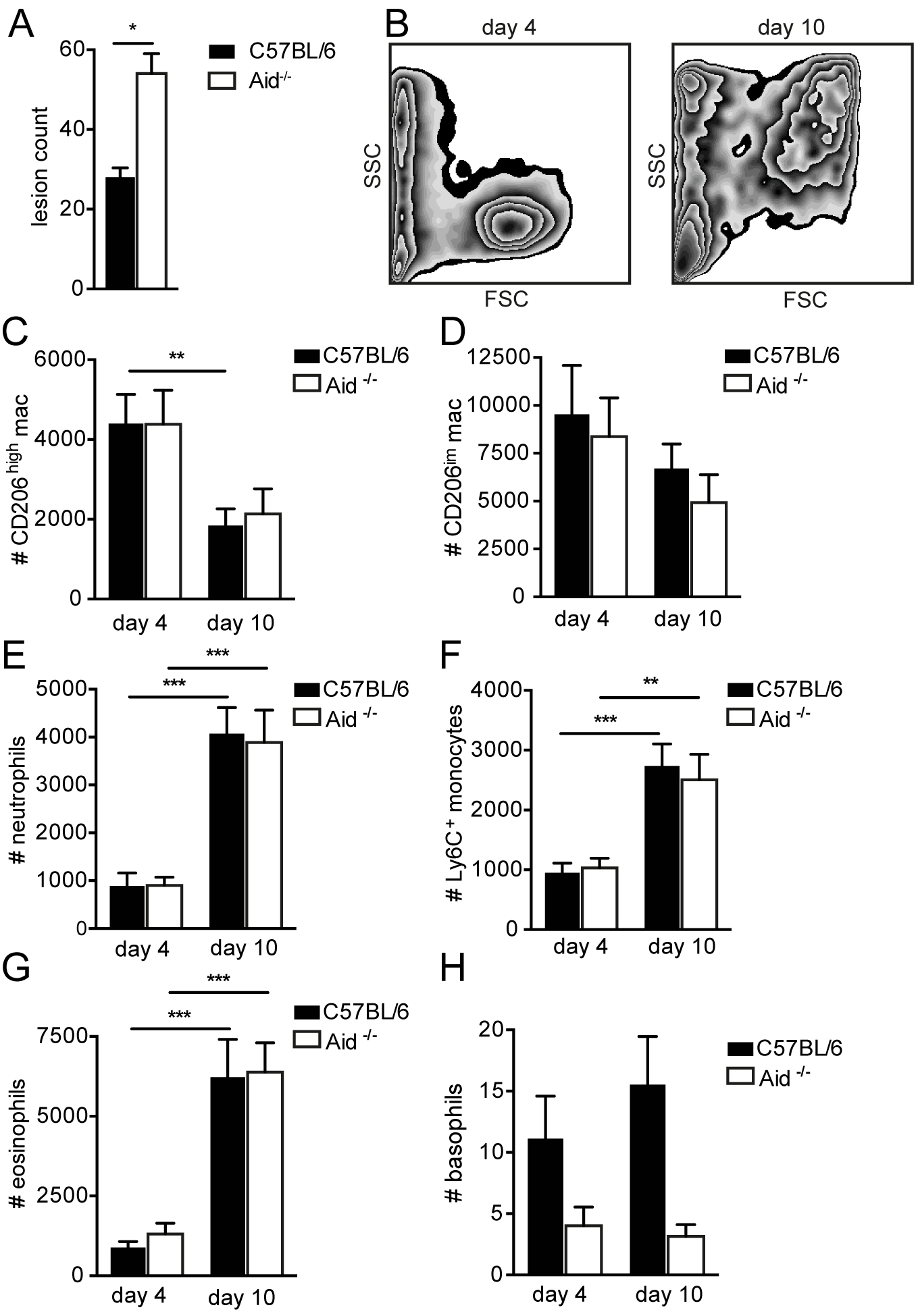

Supplement: S1 Fig — Lesions in Hpb challenge-infected C57BL/6 or Aid-/- mice were counted or dissected for cell isolation followed by flow cytometric analysis. Gating was performed as published previously [10]. (A) Intestinal lesion count at day 10 post Hpb challenge infection; (B) Representative forward side scatter plots for cells isolated from intestinal lesions of challenge-infected mice at day 4 (left) or day 10 (right); (C-H) Flow cytometric analysis of cell populations at day 4 or 10 of challenge infection in lesions from C57BL/6 or Aid-/- mice: (C) CD206high macrophages, (D) CD206intermediate macrophages, (E) Neutrophils, (F) Ly6C+ monocytes, (G) Eosinophils, (H) Basophils. Data are pooled from 2 independent experiments with 4–6 mice per group and presented as mean + SEM. (TIF) [file ppat.1004778.s001.tif]

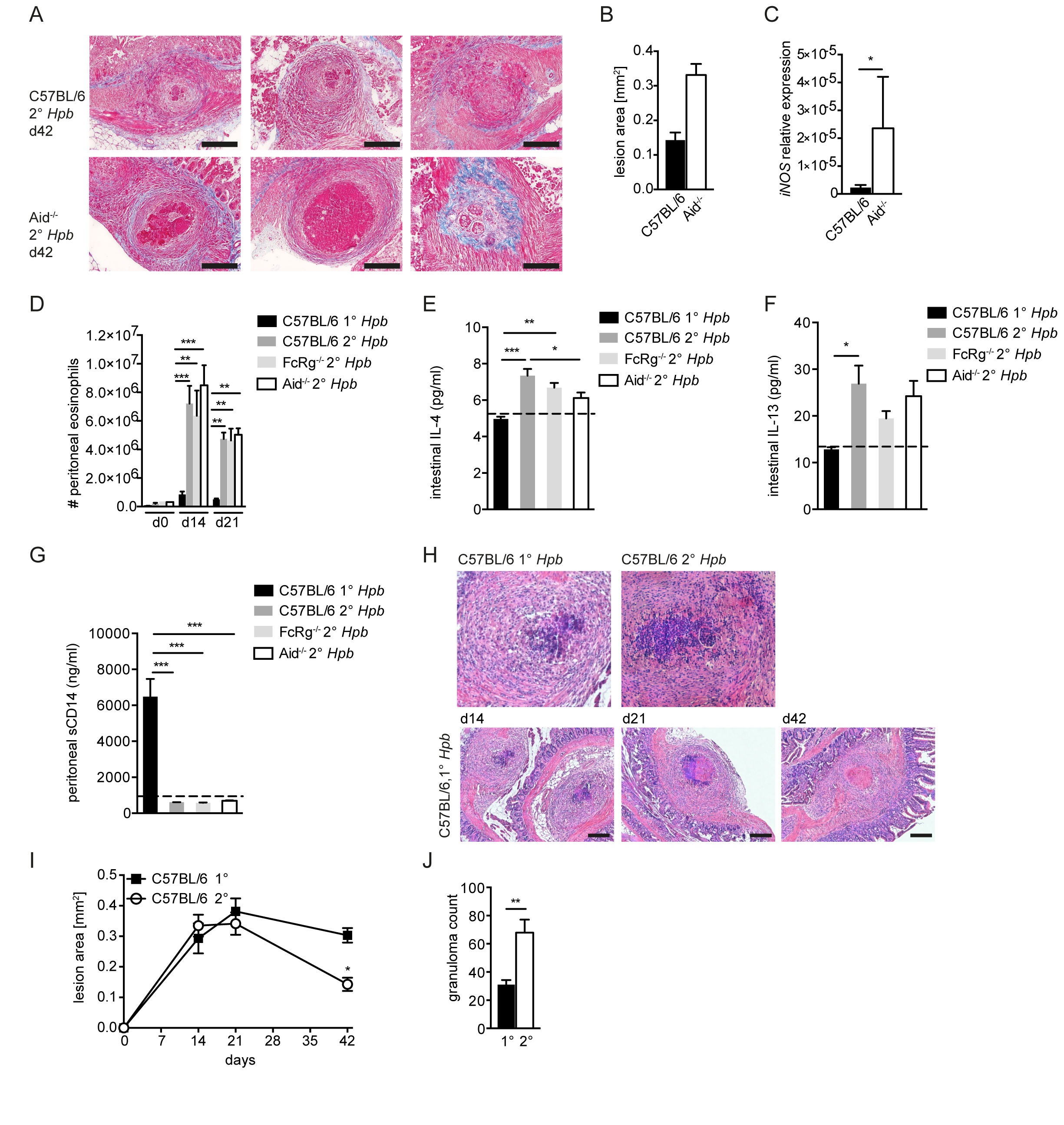

Supplement: S2 Fig — (A) Masson Trichrome staining (light pink: cytoplasm, dark pink: muscle/ keratin or necrotic tissue, blue: collagen) of intestinal lesions in challenge infected C57BL/6 WT mice or Aid-/- at day 42 post infection; (B) Quantification of lesion area of largest cross sections (day 42 p.i.) for C57BL/6 or Aid-/- mice (n = 3); (C) Expression of iNOS mRNA relative to GAPDH in total peritoneal wash cells (day 14 p.i.); (D) Number of eosinophils in the peritoneal wash from naïve, primary (1°) or secondary challenge-infected (2°) WT, Aid-/- or FcRg-/- (day 14 post infection); (E-F) Quantification of IL-4 (E) or IL-13 (F) in intestinal culture supernatants from naïve (dashed line), primary or challenge-infected WT, Aid-/- or FcRg-/- mice (day 14 post infection); (G) Quantification of soluble CD14 levels in the peritoneal wash from naïve (dashed line), primary (1°) or secondary challenge-infected (2°) WT, Aid-/- or FcRg-/- (day 14 post infection); (H) Upper panel: Representative H&E images of the centre of intestinal lesions in primary and secondary Hpb infected mice (day 14), Lower panel: Representative H&E images of lesions at day 14, 21 and 42 of primary infection (I) Quantification of lesion area during primary and secondary infection over time; (J) Enumeration of intestinal lesions (visible by eye) in the small intestine of primary or secondary Hpb infected WT mice; Data are pooled from 2 independent experiments with 3–6 mice per group and presented as mean + SEM. (TIF) [file ppat.1004778.s002.tif]

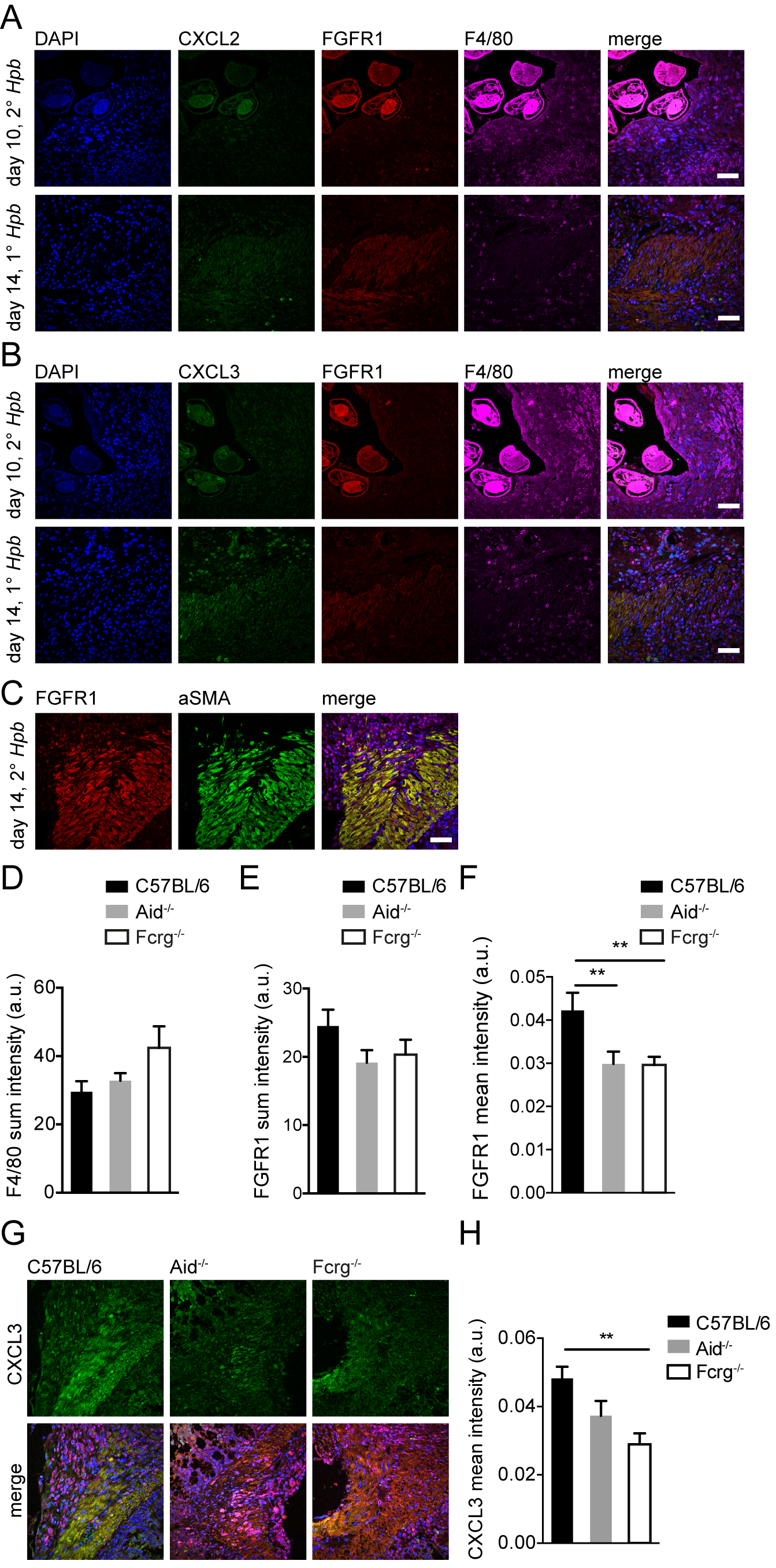

Supplement: S3 Fig — Duodenal tissues from primary or secondary Hpb infected mice were IF-stained for CXCL2 or CXCL3 together with FGFR1 and F4/80 followed by counterstaining with DAPI. (A) Upper panel: CXCL2/ FGFR1/ F4/80 staining at day 10 post challenge infection, lower panel: CXCL2/ FGFR1/ F4/80 staining at day 14 post primary infection; (B) Upper panel: CXCL3/ FGFR1/ F4/80 staining at day 10 post challenge infection, lower panel: CXCL3/ FGFR1/ F4/80 staining at day 14 post primary infection; (C) FGFR1 and aSMA staining at day 14 post challenge infection; (D) Total F4/80 intensities for the corresponding regions used for the CXCL2 quantification in Fig. 2B (see main text). (E) Total FGFR1 intensities for the corresponding regions used for the CXCL2 quantification in Fig. 2B (main text). (F) Mean FGFR1 intensities for the corresponding regions used for the CXCL2 quantification in Fig. 2B (main text). Data are pooled from 2 independent experiments with 5 mice per group and presented as mean + SEM. (G) Representative images of IF staining for CXCL3 (upper panel) or overlays of CXCL3 (green), FGFR1 (red), F4/80 (magenta) and DAPI (blue) (lower panel) in intestinal lesions from C57BL/6, Aid-/- or Fcrg-/- mice; (H) Quantification of mean CXCL3 fluorescence intensity in IF-stained intestinal lesions from C57BL/6, Aid-/- or Fcrg-/- mice; All data are pooled from 2–3 independent experiments with 3–6 mice per group and presented as mean + SEM. (TIF) [file ppat.1004778.s003.tif]

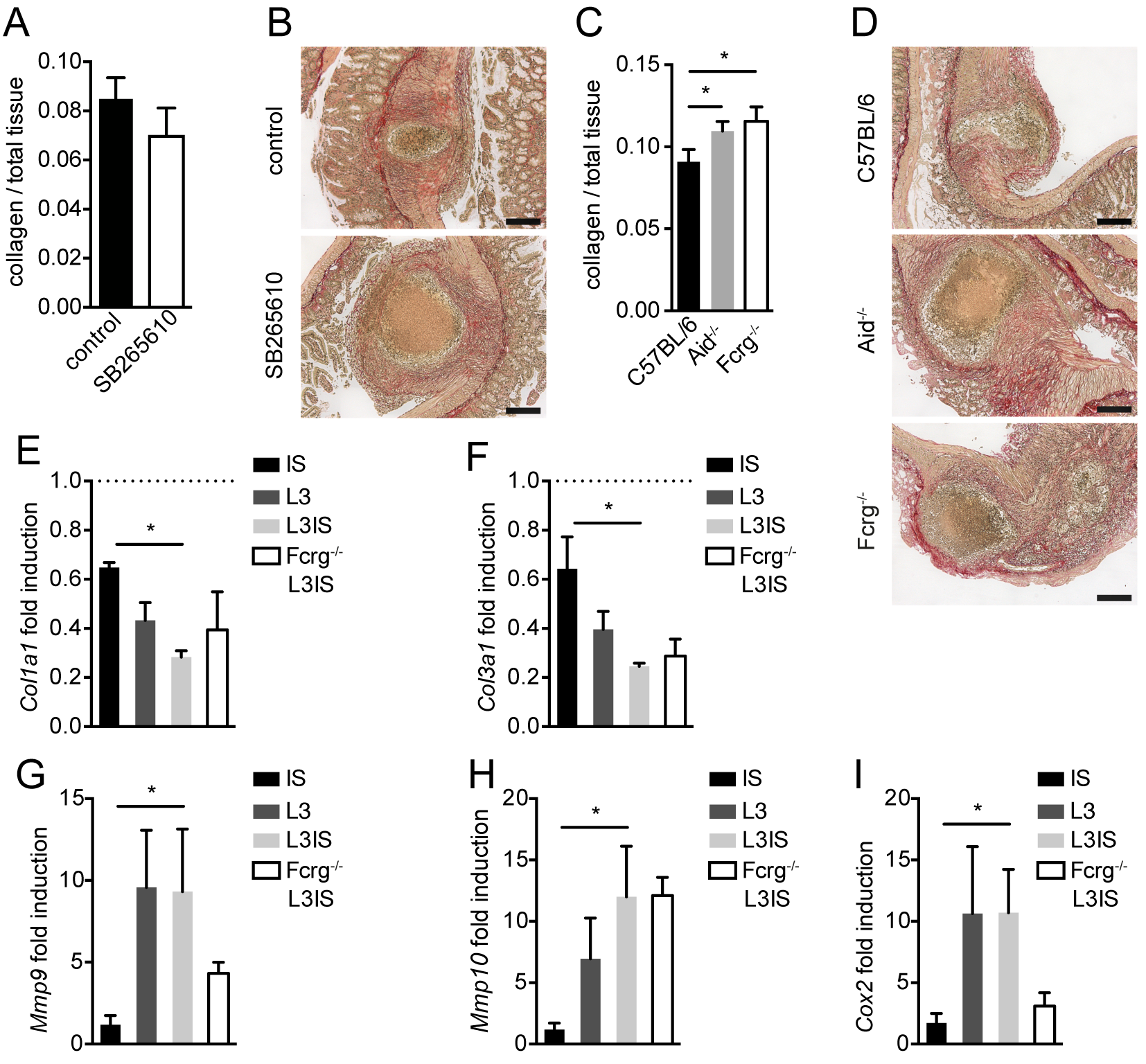

Supplement: S4 Fig — (A-D) Intestinal tissues from challenge-infected C57BL/6, Aid-/- or Fcrg-/- mice were stained for collagen with Sirius red and Sirius red staining in intestinal lesions was quantified using ImageJ; (E-I) Expression of remodeling genes in C57BL6 or Fcrg-/- MF after co-culture with IS, larvae (L3) or both (L3IS) was analyzed by qPCR and normalized to expression levels in unstimulated MF; (A) Quantification of collagen staining within intestinal lesions of untreated control mice and mice treated with the CXCR2 antagonist SB265610; (B) Representative images of Sirius red-stained lesions of control and SB265610-treated mice; (C) Quantification of collagen staining within intestinal lesions of C57BL/6, Aid-/- or Fcrg-/- mice; (D) Representative images of Sirius red-stained lesions of C57BL/6, Aid-/- or Fcrg-/- mice; (E) Fold induction of Collagen 1 A1 (Col1a1) mRNA; (F) Collagen 3 A1 (Col3a1) mRNA; (G) fold induction of matrix metalloproteinase 9 (Mmp9) mRNA; (H) fold induction of Mmp10, (I) fold induction of cyclooxygenase 2 (Cox2) mRNA in in vitro cultured intestinal MF. Data are pooled from 2 independent experiments with tissues or cells from 3–6 mice per group and presented as mean + SEM. (TIF) [file ppat.1004778.s004.tif]

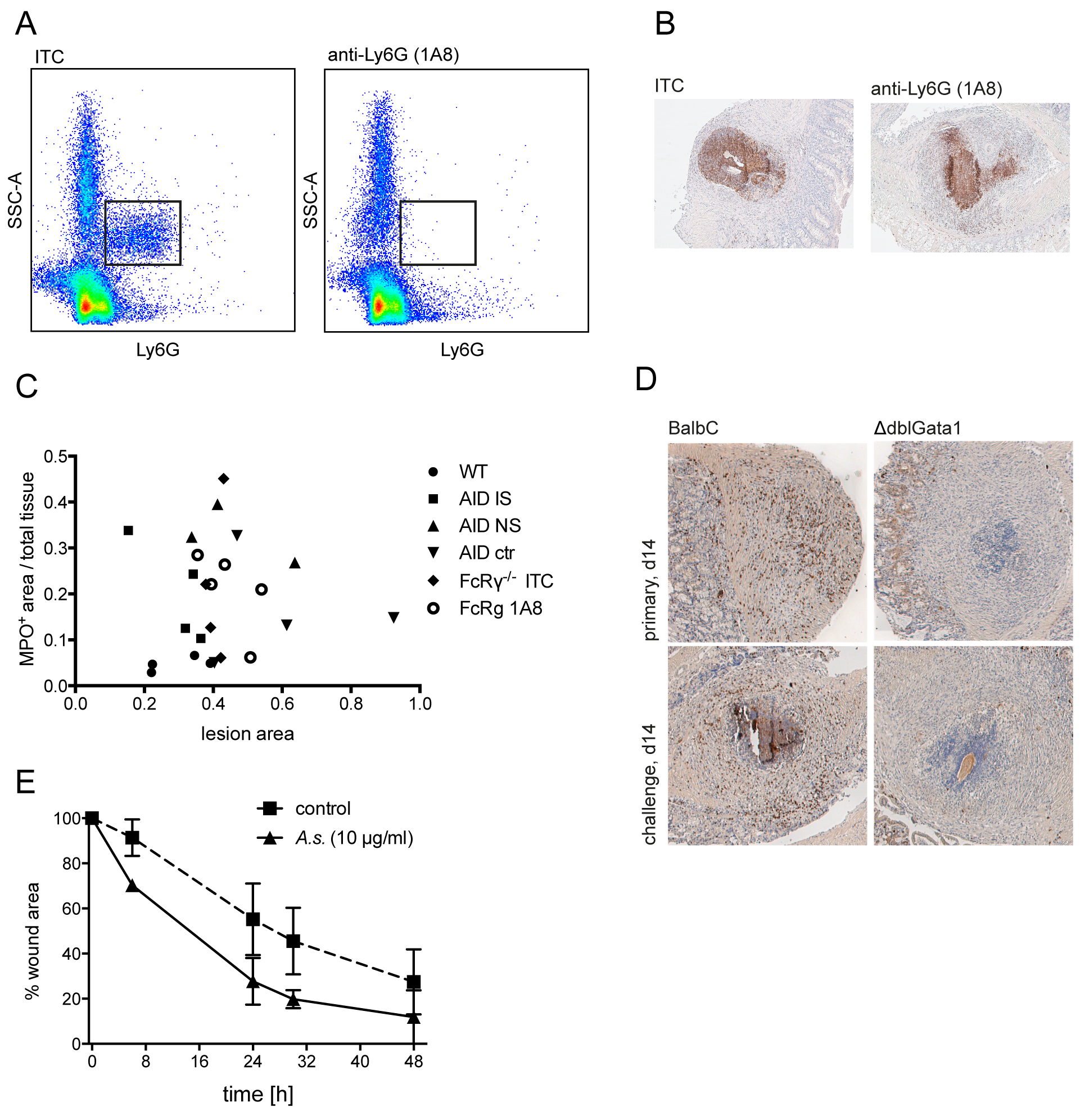

Supplement: S5 Fig — Fcrg-/- mice were treated with an anti-Ly6G antibody (1mg/day, i.p.) between day 10 and 14 of challenge infection to deplete neutrophils. (A) Neutrophil depletion in the peripheral blood of challenge-infected Fcrg-/- mice (day 14 p.i.), left: isotype control (ITC), right: anti-Ly6G (B) Small intestinal tissue sections from challenge-infected Fcrg-/- mice (ITC or Ly6G treated) were IHC-stained for MPO. (C) The size of individual lesions (in challenge-infected WT, Fcrg-/- ITC/ Ly6G, Aid-/- ctr/ NS/ IS) was plotted against the area of MPO+ cells within each lesion. (D) Representative images of IHC staining for 12/15-lipoxygenase (12/15LO) in intestinal lesions of Hpb infected BalbC or eosinophil deficient dblGata1 mice; upper panel: 12/15LO staining at day 14 of primary infection; lower panel: 12/15LO staining at day 14 of challenge infection. (E) Scratch wound closure by human myofibroblasts in the absence or presence of Ascaris suum (A.s.) products; Representative data from 2 independent experiments are shown. (TIF) [file ppat.1004778.s005.tif]

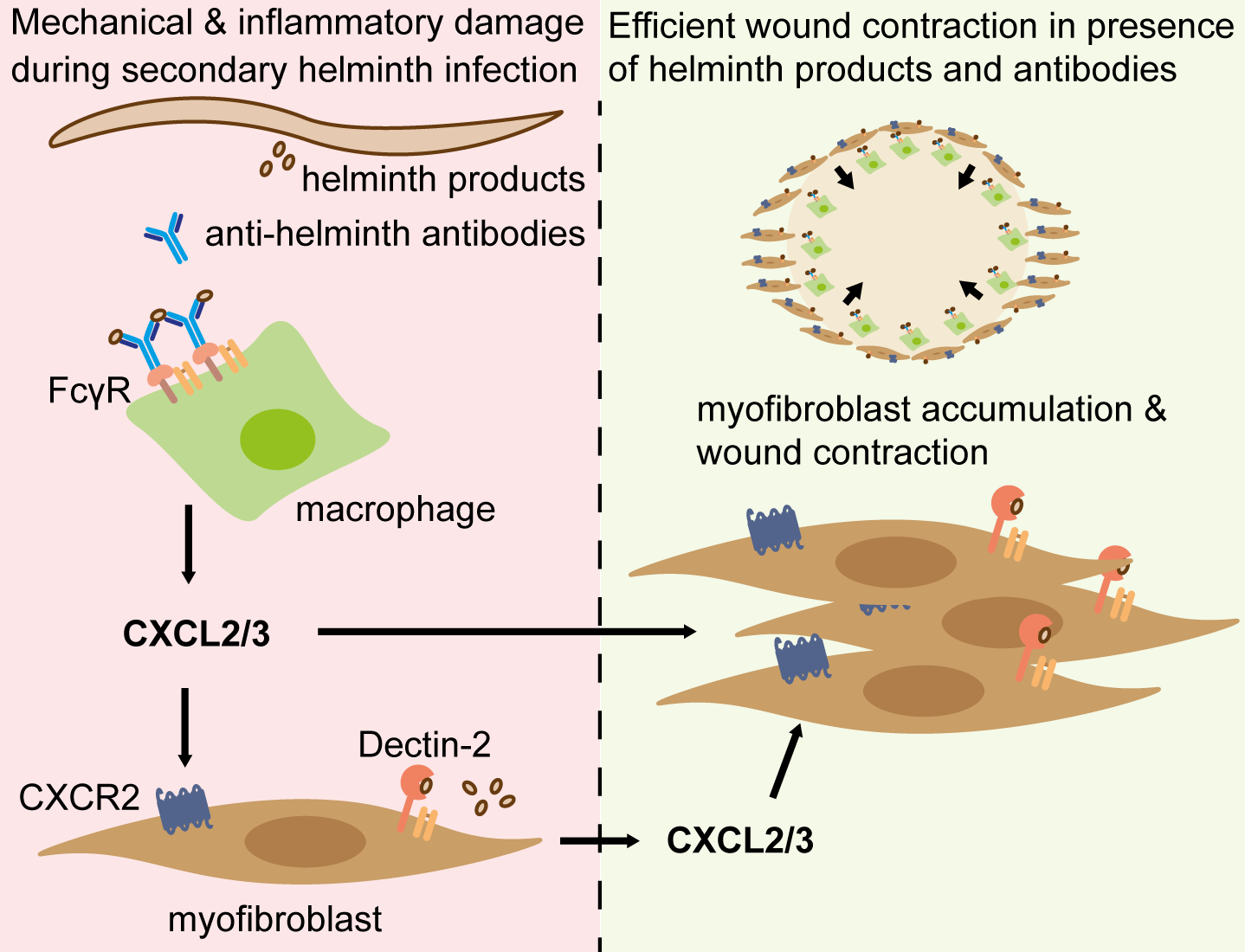

Supplement: S6 Fig — (TIF) [file ppat.1004778.s006.tif]
