# Supplementary material for: Immune Antibodies and Helminth Products Drive CXCR2-Dependent Macrophage-Myofibroblast Crosstalk to Promote Intestinal Repair
Source: PLoS Pathog. 2015 Mar 25;11(3):e1004778. doi: 10.1371/journal.ppat.1004778 (PMC4373753; doi:10.1371/journal.ppat.1004778)
Supplement: S1 Text — Detailed Materials and Methods including antibodies and primer sequences and description of 12/15-lipoxygenase as a marker for intestinal eosinophils. (DOCX) [file ppat.1004778.s009.docx]

**Supporting Information**

**Immune antibodies and helminth products drive CXCR2-dependent macrophage-myofibroblast crosstalk to promote intestinal repair**

Julia Esser-von Bieren, Beatrice Volpe, Duncan B Sutherland, Jérôme Bürgi, Sjef Verbeek, Benjamin J Marsland, Joseph F Jr Urban, Nicola L Harris

**Supplemental Results**

**12/15-Lipoxygenase is a marker for eosinophils in intestinal lesions**

As established markers for eosinophils did not produce high-quality staining within intestinal lesions, possibly due to abundant necrosis, we searched for a more reliable and quantifiable marker of intestinal eosinophils. Eosinophils are known to highly express enzymes for eicosanoid biosynthesis, including 12/15-lipoxygenase (12/15LO) (1). In order to confirm that 12/15-LO specifically stains eosinophils in intestinal lesions, we made use of eosinophil deficient dblGata1 mice (2). Eosinophil lineage ablated mice showed a complete absence of 12/15LO staining in intestinal lesions during both primary and challenge *Hpb* infection (Fig. S4).

**Supporting Methods**

**Mice**

C57BL/6, BALB/c, Aid^-/-^ (3), Fcrg-chain^-/-^ (4) and dblGata 1 (2) were bred and maintained under specific pathogen free conditions at the EPFL. Dectin-2^-/-^ mice (5) were bred and maintained under specific pathogen free conditions at the University of Lausanne (UNIL), Epalinges, Switzerland. All genetically engineered mice were backcrossed for at least 10 generations to the appropriate wildtype strain (C57BL/6 for Aid^-/-^, Fcrg-chain^-/-^ and dectin2^-/-^, BalbC for dblGata1).

**Infection and parasitology**

Mice were infected with 200 L3 larvae by oral gavage as described elsewhere. Worms were cleared by treatment with two courses of Cobantril (Interdelta - Givisiez, Fribourg, Switzerland) 28 days after primary infection. 14 days later mice were re-infected with 200 L3 larvae. Small intestines were harvested at days 4-21 post secondary challenge infection for preparation of “Swiss rolls”, organ culture or lamina propria isolation. To avoid any impact of high worm burdens in Aid^-/-^ or Fcrg^-/-^ mice on the repair process, mice were additionally treated with cobantril at days 8 and 10 post challenge infection. The absence of worms in the small intestine was confirmed at necropsy in all experiments. In addition, egg counts were performed in some experiments to confirm the efficacy of deworming after primary infection. Lesions or adult worms were counted under a stereomicroscope on longitudinally opened small intestines of challenge-infected mice.

**Challenge infections of pigs with *A. suum***

Cross-bred pigs were maintained at the Beltsville Agricultural Research Center in Beltsville, Maryland, USA and inoculated with infective *A. suum* eggs as described in (6). Briefly, three mixed-sex seven weeks old pigs were inoculated per os with 1,000 infective *A. suum* eggs every other day four times and three other naïve pigs were untreated and served as uninfected control pigs. All pigs were anthelmintic drug-treated with fenbendazole (10mg/kg body weight) nine weeks after the first inoculation, rested for two weeks, and challenged infected with 10,000 *A. suum* eggs. The pigs were bled for serum isolation at the time of challenge infection and 14 days later.

**In vitro cultures of human myofibroblasts**

Primary human fibroblasts were obtained from explant of gingival tissues and grown at 37°C 5% CO_2_ in DMEM medium (Gibco, Carlsbad, CA, USA) supplemented with 10% fetal bovine serum (GE Healtcare) and 1% penicillin and streptomycin (Gibco, Carlsbad, CA, USA). Myofibroblast differentiation was induced by exogenously adding 5ng/ml TGFβ1 (Sigma-Aldrich, St Louis, MO) once for 3 days in DMEM supplemented with 0.5% FBS. All experiments were performed with cells after a maximum of 15 passages. Differentiation into MF was verified by analyzing the upregulation of aSMA by immunofluorescence staining.

**Antibodies used for flow cytometry and histology**

The following monoclonal antibodies were used for surface staining for flow cytometric analysis: anti-CD45 Alexa Fluor 700, anti-F4/80 FITC, anti-Ly6G PE-Cy7, anti-CD4 Pacific Blue, (all from BioLegend, San Diego, CA), anti-FceR PE, anti-IgE PE, anti-Siglec F PE (all from BD Biosciences, Franklin Lakes, NJ). Cells from granuloma were stained with Live Dead stain Aqua (Lifetechnologies, Zug, Switzerland) before proceeding to surface stain.

The following primary and secondary antibodies were used for histological stainings: polyclonal rabbit anti-mouse CXCL2, CXCL3 (Biorbyt, Cambridge, UK), 12-lipoxygenase, α-smooth muscle actin (Abcam, Cambridge, UK), rat anti-mouse F4/80 (AbD Serotec, Kidlington, UK), goat anti-mouse FGFR1 (Aviva Systems Biology, San Diego, CA) Alexa Fluor 488 conjugated donkey anti-rat IgG, Alexa Fluor 647 conjugated donkey anti-rabbit IgG and Alexa Fluor 568 conjugated donkey anti-goat (Lifetechnologies, Zug, Switzerland). Tissues were counterstained with DAPI (Lifetechnologies, Zug, Switzerland).

**Microscopy and image analysis**

Stained tissue sections were imaged with a Leica DM5500 widefield microscope with a HCX PL FLUOTAR (5x/0.15 NA) objective or an inverted point scanning confocal microscope (Zeiss LSM 710) with a Plan-Apochromat (63x/1.4 NA or 40x/1.3 NA) objective and DAB staining or fluorescence pixel intensities were analyzed using Fiji or CellProfiler respectively (7).

**Supporting Tables**

| **Gene name** | **Sequence forward primer 5’-3’** | **Sequence reverse primer 5’-3’** |
| --- | --- | --- |
| *Cxcl2* | TCAAGGGCGGTCAAAAAGTT | TCCTCCTTTCCAGGTCAGTTA |
| *Cxcl3* | CCATCCAGAGCTTGACGGTGAC | GGCTCAGCTGGACTTGCCGCTC |
| *gapdh* | GGGTGTGAACCACGAGAAAT | CCTTCCACAATGCCAAAGTT |
| *Col1a1* | ACGGCTGCACGAGTCACAC | GGCAGGCGGGAGGTCTT |
| *Col3A1* | GTTCTAGAGGATGGCTGTACTAAACACA | TTGCCTTGCGTGTTTGATATTC |
| *Mmp9* | CTAAAGGCCATTCGAACACC | CGCGGCAAGTCTTCAGAGTA |
| *Mmp10* | CACCTGGCCCTGGATTTTATGG | GCTGGGCTTGTGGAGAACCTGTA |
| *Cox2* | GCATTCTTTGCCCAGCACT | AAAGGCGCAGTTTACGCTGT |

**Table S1: Primer sequences used for quantitative real-time PCR**

**Supplemental References**

1. Nakamura M et al. Immunocytochemical localization of platelet-type arachidonate 12-lipoxygenase in mouse blood cells. *J Histochem Cytochem Off J Histochem Soc*. 1995;43(3):237–244.

2. Yu C et al. Targeted deletion of a high-affinity GATA-binding site in the GATA-1 promoter leads to selective loss of the eosinophil lineage in vivo. *J Exp Med*. 2002;195(11):1387–1395.

3. Muramatsu M et al. Class switch recombination and hypermutation require activation-induced cytidine deaminase (AID), a potential RNA editing enzyme. *Cell*. 2000;102(5):553–563.

4. Takai T, Li M, Sylvestre D, Clynes R, Ravetch JV. FcR gamma chain deletion results in pleiotrophic effector cell defects. *Cell*. 1994;76(3):519–529.

5. Saijo S et al. Dectin-2 recognition of alpha-mannans and induction of Th17 cell differentiation is essential for host defense against Candida albicans. *Immunity*. 2010;32(5):681–691.

6. Urban JF et al. Bacillus thuringiensis-derived Cry5B has potent anthelmintic activity against Ascaris suum. *PLoS Negl Trop Dis*. 2013;7(6):e2263.

7. Esser-von Bieren J et al. Antibodies Trap Tissue Migrating Helminth Larvae and Prevent Tissue Damage by Driving IL-4Rα-Independent Alternative Differentiation of Macrophages. *PLoS Pathog*. 2013;9(11):e1003771.
